# Aerosol and splatter generation with rotary handpieces used in restorative and orthodontic dentistry: a systematic review

**DOI:** 10.1038/s41405-022-00118-4

**Published:** 2022-09-06

**Authors:** Waraf Al-yaseen, Rhiannon Jones, Scott McGregor, William Wade, Jennifer Gallagher, Rebecca Harris, Ilona Johnson, Sukriti KC, Mark Robertson, Nicola Innes

**Affiliations:** 1 Paediatric Dentistry Department, School of Dentistry, College of Biomedical and Life Sciences, Cardiff University, Heath Park, Cardiff, CF14 4XY UK; 2 Education, Scholarship and Innovation, School of Dentistry, College of Biomedical and Life Sciences, Cardiff University Heath Park, Cardiff, CF14 4XY UK; 3 Service Delivery Division, Library and Learning Centre, University of Dundee, Dundee, DD1 4HR UK; 4 Centre for Host-Microbiome Interactions, Faculty of Dentistry, Oral & Craniofacial Sciences, King’s College London, Guy’s Campus, London, SE1 9RT UK; 5 Dean for International Affairs | Newland-Pedley Professor of Oral Health Strategy | Hon Consultant in Dental Public Health, London, UK; 6 Faculty of Dentistry, Oral & Craniofacial Sciences | King’s College London | Bessemer Rd | London, SE5 9RS London, UK; 7 Deputy Chief Dental Officer for England. Professor/Honorary Consultant in Dental Public Health, the University of Liverpool, Room 124, First Floor, Block B Waterhouse Building, 1-5 Brownlow Street, Liverpool, L69 3GL England; 8 Health Improvement Division, Public Health Wales, Capital Quarter, Tyndall Street, Cardiff, CF10 4BZ UK; 9 Visiting Research Associate- Faculty of Dentistry, Oral & Craniofacial Sciences, King’s College London, Bessemer Rd, London, SE5 9RS UK; 10 PhD Candidate- School of Public Health, Imperial College London, The Reynolds Building, St. Dunstan’s Road, London, W6 8RP UK; 11 Child Dental and Oral Health, School of Dentistry, University of Dundee, Park Place, Dundee, DD1 4HR UK; 12 School of Dentistry, College of Biomedical and Life Sciences, Cardiff University, Heath Park, Cardiff, CF14 4XY UK

**Keywords:** Dental equipment, Infection control in dentistry

## Abstract

**Introduction:**

The COVID-19 pandemic has caused major disruptions in dental care globally, in part due to the potential for contaminated aerosol to be generated by dental activities. This systematic review assesses the literature for changes in aerosol-contamination levels when rotary instruments are used, (1) as distance increases from patient’s mouth; (2) as time passes after the procedure; and (3) when using different types of handpieces.

**Methods:**

The review methods and reporting are in line with PRISMA statements. A structured search was conducted over five platforms (September 2021). Studies were assessed independently by two reviewers. To be eligible studies had to assess changes in levels of aerosol contamination over different distances, and time points, with rotary hand instruments. Studies’ methodologies and the sensitivity of the contamination-measurement approaches were evaluated. Results are presented descriptively.

**Results:**

From 422 papers identified, 23 studies were eligible. All investigated restorative procedures using rotary instruments and one study additionally looked at orthodontic bracket adhesive material removal. The results suggest contamination is significantly reduced over time and distance. However, for almost all studies that investigated these two factors, the sizes of the contaminated particles were not considered, and there were inconclusive findings regarding whether electric-driven handpieces generate lower levels of contaminated particles.

**Conclusion:**

Aerosol contamination levels reduce as distances, and post-procedure times increase. However, there was sparce and inconsistent evidence on the clearing time and no conclusions could be drawn. High-speed handpieces produce significantly higher levels of contamination than slow-speed ones, and to a lesser extent, micro-motor handpieces. However, when micro-motor handpieces were used with water, the contamination levels rose and were similar to high-speed handpiece contamination levels.

## Background

Prevention of transmission of respiratory and blood-borne infections has long been a concern for the dental profession [1]. Aerosols produced during dental procedures are of particular interest and importance to the dental profession. Rotary handpiece instruments have been implicated because they often incorporate air and/or water as part of their working mechanisms to maximise efficiency and reduce clinical time. The generation of aerosols (suspensions of particles <5 µm diameter), droplets (particulate matter >5 µm) and splatter (or spatter) (particulate matter >50 µm) [2], during their use, means there is potential for blood borne, respiratory and salivary pathogenic microorganisms to be held in the air for some time and carried for some distance during and after procedures. Theoretically, these can then contaminate the dental team, patients, and surrounding surfaces, increasing transmission rates.

The COVID-19 pandemic highlighted fresh concerns regarding dental aerosol contamination levels with the potential transmissibility of SARS-CoV-2 between staff and patients in both directions. This led to complete interruption in routine dental services in most parts of the world. The service disruption, ongoing reduction in service capacity associated with social distancing, and enhanced personal protective equipment (PPE) [3] together with requiring a “fallow time” post-procedure (a clearing period after any “aerosol-generating procedures” where the dental clinic is kept unoccupied, before any decontamination and undertaking the next procedure) have contributed to a substantial backlog of patient care in the UK. At the beginning of the SARS-CoV-2 pandemic, systematic reviews collated scientific evidence to inform the profession and policy makers of likely transmission risk. However, only indirect evidence was available (i.e., no studies addressed the actual transmission rates of disease between patients and dentists in either direction). The indirect evidence came from studies using methods to visualise the extent of spread of bio-aerosols and splatter. Attempts to synthesise the data from these studies and draw conclusions were hampered by the varied methodologies of the studies and the (often unquantifiable) assumptions made [4]. To inform guidelines on fallow times following procedures and suitable PPE, further primary research studies have been carried out. With the gap in direct evidence of actual risk of COVID transmission through dental procedures, the guidelines based on indirect evidence have been crucial in informing protocols, thus governing the safe resumption of dental services [5]. They have been updated regularly as more evidence has become available. Efforts to find mitigations to reduce aerosol generation have not only covered ways of reducing them after they have been generated but also techniques that avoid producing them in the first place, and mechanisms that reduce their generation, such as electric micro-motor handpieces that do not use an influx of air influx to rotate dental burs.

The impact that fallow time has on reducing the number of dental appointments makes understanding distances and time involved in the spread and settle of aerosol and droplets important to understand.

The aim of this review is to investigate contamination levels of the dental environment for different types of handpieces used in restorative and orthodontic procedures routinely carried out in the dental surgery, over distance and time and to incorporate new evidence on the relative aerosol and splatter generated during use of electric micromotors, air-turbine handpieces and slow speed handpieces.

This systematic review is part of a wider body of research conducted to improve understanding of dentistry-associated aerosol generation and dental environment contamination. It builds on the findings of a systematic review carried out in 2020–2021 [6] and brings more insight into evidence around aerosol generation due to the use of rotary handpieces, complementing the findings from the two other focussed reviews that have been published exploring the level and pattern of aerosol contamination generated during oral surgery [7], and periodontal-related [8], procedures. It should be noted that the term aerosol is used throughout the review but there is often no distinction in the research papers between aerosol, droplet and splatter being measured, and a lack of accuracy and clarity in the terminology used.

The objectives of the review are to assess changes in aerosol and splatter contamination levels and spread patterns when rotary instruments are used:

- as the distance increases from the procedure location;
- over time following the procedure; and
- for different types of rotary handpieces.

## Methods

This review is an update of a wider systematic review of AGPs in dentistry undertaken in 2020 (protocol registration ID: CRD42020193058) [9], and it focusses on rotary instruments used in restorative and orthodontic procedures. It has been conducted and presented in line with PRISMA guidelines [10].

### Literature search

A search of the literature up to 16th September 2021 was performed in Medline (OVID), Embase (OVID), Cochrane Central Register of Controlled Trials, Scopus, Web of Science, and LILACS databases. All search strategies and search terms were developed by an experienced Information Technologist and Librarian. The search strategy comprised controlled vocabulary, such as the National Library of Medicine’s MeSH (Medical Subject Headings) and keywords (Box 1 details the search strategy) [11].

Box 1 Search strategy

1. Dental Care/
2. Dental Clinics/
3. Dental Offices/
4. Dental Equipment/
5. Dental Instruments/
6. Dentistry/
7. Dentists/
8. Dental Hygienists/
9. Dental surger$.mp.
10. Dental practice$.mp.
11. Dental clinic$.mp.
12. Dental Scaling/
13. Dental Plaque/
14. Restorative.mp.
15. Orthodontic$.mp.
16. Ultrasonic.mp.
17. Ultrasound.mp.
18. Instrumentation.mp.
19. Handpiece$.mp.
20. Turbine.mp.
21. Drill.mp.
22. “Air line”.mp.
23. “Water line”.mp.
24. “Air polishing”.mp.
25. “Air abrasion”.mp.
26. Extraction$.mp.
27. Cavitation.mp.
28. Preparation.mp.
29. Filling$.mp.
30. or/14–29
31. Dent$.mp.
32. 30 and 31
33. 1 or 2 or 3 or 4 or 5 or 6 or 7 or 8 or 9 or 10 or 11 or 12 or 13 or 32
34. Cross Infection/
35. Coronavirus Infections/
36. Disinfection/
37. Infectious Disease Transmission, Patient-to-Professional/
38. Occupational Diseases/
39. Occupational Exposure/
40. Infection Control/
41. Infection Control, Dental/
42. Risk Factors/
43. Risk Management/
44. Particulate Matter/
45. Air Pollutants, Occupational/
46. Air Pollution, Indoor/
47. Blood-Borne Pathogens/
48. Spatter.mp.
49. Saliva.mp.
50. Blood particles.mp.
51. Blood borne pathogens.mp.
52. Nosocomial.mp.
53. Contamination.mp.
54. Viruses/
55. Air Microbiology/
56. Viral.mp.
57. Viridae.mp.
58. Droplets.mp.
59. “Droplet nuclei”.mp.
60. Coughing.mp.
61. Gag$.mp.
62. sneez$.mp.
63. or/34–62
64. Aerosols/
65. Bioaerosol$.mp.
66. Bio-aerosol$.mp.
67. “aerosol transmission”.mp.
68. “aerosolised transmission”.mp.
69. “aerosolized transmission”.mp.
70. “aerosol generating procedures”.mp.
71. Aerosol$.mp.
72. “bio-aerosol generating procedures”.mp.
73. “inhalation transmission”.mp.
74. “contact transmission”.mp.
75. “nosocomial transmission”.mp.
76. emissions.mp.
77. or/ 64–76
78. 33 and 63 and 77

### Inclusion and exclusion criteria

The review included observational primary research data and experimental studies where baseline data or comparison data were derived from conducting dental procedures under routine conditions, without using aerosol reduction measures. Descriptive, secondary research data, and studies that did not explore aerosol generated from a standard dental procedure (i.e., without using approaches that alter the level/extent of the generated aerosol) were excluded. The review included all types of dental setting with any level of care (primary/secondary/tertiary), simulated clinical settings with patients, or mannequins. Studies had to involve an operator in a dentist’s position with or without a dental assistant, carrying out a dental procedure or simulated dental procedure, using a dental handpiece. For this study, rotary instruments included air-driven handpieces used for restorative procedures regardless of their shape (e.g., straight or contra-angle) and speed and mechanism of action. Handpieces were considered as high-speed if they operated within the range of 200,000–400,000 revolution per minutes (rpm), and as low-speed handpieces if they operated in the range of 5000 to 40,000 rpm. Their mechanism of action is mainly through air-driven rotation, where air is forced over the vanes or blades inside the handpiece, causing them to spin. Some modern handpieces are electrically driven, and these purportedly generate less aerosol, and no air pressure is needed to operate the handpiece. For the electrically driven handpieces, we assumed there was no water spray used in the handpieces unless it was explicitly stated that there was. Studies had to include data on changes in contamination levels with aerosol generation related to (1) time and/or (2) distance and/or (3) the use of different types of handpieces. Studies not meeting the inclusion criteria were excluded.

Although the search was not limited by language, only articles published in English or Chinese were selected. We included studies in Chinese as we anticipated there might be an increase in this research area in Chinese-speaking countries in response to the pandemic. As for the outcome, studies had to explore dental aerosol contamination for three specific dimensions: changes in its level in distance, time, and the difference in contamination when using different types of rotary high pieces.

### Study selection

Studies captured by the search strategy were imported into Rayyan™(https://www.rayyan.ai/) [12], an online platform for title and abstract reviewing. Two reviewers independently screened the titles and abstracts. Full texts were retrieved for all citations considered to fit the inclusion criteria by either reviewer. Two reviewers then independently reviewed the full-text articles and selected studies against the inclusion/exclusion criteria and discrepancies between reviewers were resolved by consensus. An independent third reviewer was consulted where a decision could not be reached. The bibliographies of the included papers were hand-searched to look for potential studies meeting the criteria. Corresponding authors were contacted when more information than was presented in the published paper, was required. Publishing journals were directly contacted when the full text was not electronically available.

### Data extraction and analysis

Included studies were divided amongst the review team for data extraction. The process was conducted by one reviewer and verified by a second reviewer using a pre-designed data extraction form (previously piloted for the initial review). The following data fields were extracted: study characteristics (information about the location of the study, date of publication, the setting); methodology (the procedure conducted, the instrument used, the rotation speed of the handpiece used); and results (changes in the level and extent of aerosol while using the rotary air motor over distance and time). Any disagreements between reviewers were resolved by discussion to reach a decision. The data are presented descriptively.

### Quality assessment

There were no appropriate tools that had measured the quality of studies investigating aerosol generation or contamination evident in the published literature. As there was no pre-prepared and validated tool for quality assessment, we build on previous similar areas to create one. Quality assessment categories were found after reviewing the literature and the assessment criteria were established after discussion on the appropriateness of these with one of the authors who is an experienced oral microbiologist and researcher (WW) [13, 14]. The quality of the included studies was assessed against key criteria reflecting the risk of bias (industry-funded, conflict of interest), quality of the study methodology (sample size calculation, control, and outcome measurements), reporting quality (description of the procedure and equipment’s used), and applicability (relevancy to modern dentistry) [7]. Two independent reviewers conducted the assessment using a traffic light system for scoring study quality. Red meant that the study did not meet the expected standard for that domain. Yellow: the study partially met the expected standards, or no details were mentioned; and green: the study met expected standards. A full description of the quality assessment has been previously described [7] and can be found in the review protocol [9].

### Sensitivity of the contamination measurement

Contamination measurement methods and tools used in the studies were evaluated and ranked according to their sensitivity to detect aerosol and droplets. The assessment was carried out in duplicate by two reviewers (WA and NI). The studies were categorised by the contamination sampled: microbial, blood, non-microbial/non-blood contamination Three scores were used to assess the sensitivity of the contamination measurements: Low, Moderate, and High. The definitions for each score, for each category can be found at the review protocol [9].

### Data handling and reporting

Included studies were grouped according to their outcome, i.e., distance, time, and types of handpieces. For each outcome, a further subgrouping was reported on which was related to the contamination measurement method (settle plate, air sampling, filtered papers). Each study design was summarised in a table that included handpiece speed, and selected outcome measure. The related results were presented in further tables. Existing evidence was summarised in the supporting narrative, and knowledge gaps were identified. Meta-analyses were planned if the included studies were sufficiently homogenous in terms of the study design, methodology, and outcomes. The recommendations of the PRISMA statement for the report of this systematic review were followed [10].

## Results

### Study selection

**Fig. 1:**
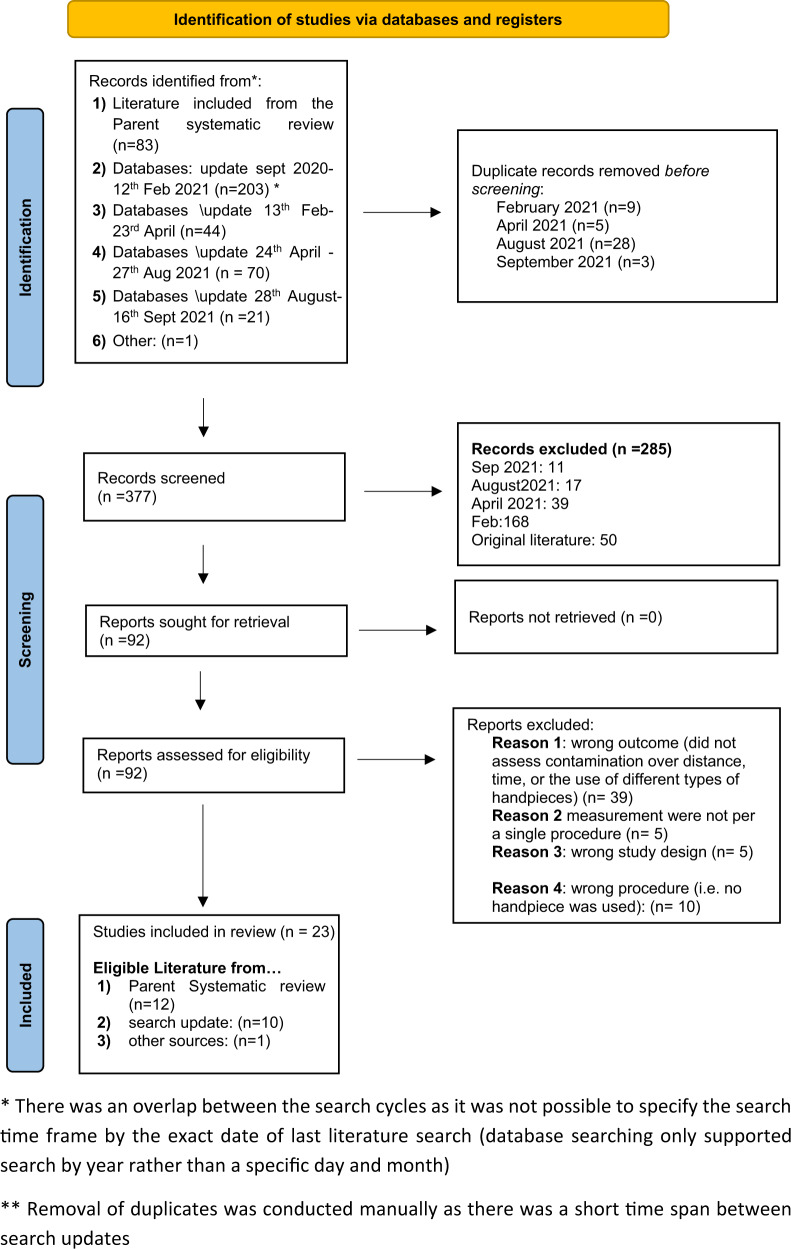
PRISMA Flow diagram of the study [10]. PRISMA diagram, flowchart demonstrating the results of searches and study selection.

Studies were pooled from two main sources. First, the studies that were included in the parent systematic review [7]; and second, the systematic database search which was updated four times due to the fast turnover of the publications related to this topic (see Fig. 1). Additional database searches and the previously identified papers from our original study were combined and one additional article was found from other sources, resulting in 422 papers. After the removal of duplicates, 377 remained. Following title and abstract screening for eligibility, the full text of 92 articles were retrieved and assessed. This included the studies from the parent systematic review [7] which were considered against the specific inclusion criteria for this review. From these, 69 were excluded for the following reasons: wrong outcome (e.g., assessment of environmental or waterline contamination) (*n* = 39); measurements were not associated with single procedure (*n* = 5), wrong study design (*n* = 5), wrong procedure (i.e no handpiece was used) (*n* = 10), and duplication (*n* = 10) and 23 studies were included in the final dataset. We contacted two authors for additional information on their studies [15, 16].

### Study characteristics

There were 23 papers and studies which met the inclusion criteria [15–36]. These were carried out between 1964 to 2021, across 12 countries (USA *n* = 6, UK *n* = 7, Saudi Arabia *n* = 1, Australia n = 1, Canada *n* = 1, Egypt n = 1, Finland *n* = 1, India *n* = 1, Italy *n* = 1, Japan *n* = 1, Lithuania *n* = 1, Portugal *n* = 1).

Most studies were observational in design (*n* = 20) [15–28, 31–34]. Five investigating the efficacy of an intervention [15, 17, 30, 33, 34], and three studies included both methodologies [17, 33, 34]. For further details, see the online Supplementary Information.

### Study settings

The studies are described in a variety of ways. Table 1 shows them grouped by setting (clinical skills training laboratory, single surgery, open plan clinic) and treatment conditions (simulated or actual treatment with a patient or a mannequin). Some studies had multiple settings and methodologies.

**Table 1:** Study setting details.

Study	Setting			Treatment		
	Clinical skills training laboratory	Single surgery	Open plan clinic	Treatment on patient	Simulated treatment on patient	Mannequin
Belting 1964 [20]		X			X (patients TB +ve)	
Hausler 1966 [23]		X				X
Larato 1966 [25]		X		X		
Miller 1971 [28]		X		X		
Samaranayake 1989 [32]		X		X		
Bentley 1994 [21]		X		X		X
Grenier 1995 [22]		X		X		
Tag El-Din 1997 [33]		X		X		
Purohit 2009 [30]				X		
Rautemaa 2006 [31]		X		X		
Yamada 2011 [34]		X		X		
Manarte-Monteiro 2013 [27]		X		X		
Ionescu 2020 [24]		X				X
Nulty 2020 [29]		X				X
Ahmed 2021 [37]	X					X
Allison 2021(a) [18]	X					X
Allison 2021(b) [19]			X			X
Allison 2021(c) [35]	X					X
Grzech-Lesniak 2021 [36]		X				X
Han 2021 [16]		X				X
Holliday 2021 [15]	X		X			X
Llandro 2021 [26]	X					X
Shahdad 2021 [17]		X	X			X

Most studies (*n* = 16) were carried out in a clinical setting. Of these, three were in general dental practice [16, 23, 24] and 12 were in a dental hospital.

Five studies were performed in laboratories [15, 18, 19, 26]. The setting could not be determined for two studies [20, 28].

For the 16 clinical setting studies, most were set in single surgeries (*n* = 13), five were in multiple chair clinics and three were in both types of settings (see Table 1).

### Dental procedures

All 23 studies investigated restorative procedures involving tooth preparation (fixed prosthodontic tooth preparation and cavity preparation) using rotary instruments and one study additionally looked at orthodontic bracket adhesive material removal. The following details the types of procedures:

- Tooth preparation on a patient (clinical setting) *n* = 9 [22, 25, 27, 28, 30–34].
- Simulated tooth preparation on a patient e.g., use of high-speed water spray without tooth contact (clinical setting) *n* = 1 [20].
- Tooth preparation on a mannikin head in a dental clinic or dental simulation laboratory setting (*n* = 9) [15, 17–19, 21, 23, 24, 26].
- Tooth preparation on a mannikin head/ typodont in a laboratory setting (*n* = 1) [16].
- Removal of adhesive material following orthodontic bracket removal (*n* = 1) [26].

### Outcomes and outcome measures

The studies explored changes in the levels of aerosol, droplets, and splatter contamination: (1) across different distances from the dental procedure location; (2) over time following the procedure; and (3) with use of three different types of rotary handpieces (high-speed, micromotor, and slow speed). These outcomes were measured through:

- bacterial colony forming units (CFUs) per a surface area (e.g., cm^2^) or a plate, or volume of air. CFUs were obtained by conducting a microbial assessment, in which settle plates or air sampling were used to collect the samples. The samples were then incubated either aerobically or anaerobically to allow the bacteria to grow. [20–25, 27, 28, 30–33].
- blood spots located on a filter placed on an air sampler while conducting a restorative procedure on a patient [34].
- fluorescein-stained aerosol/droplets generated during the dental procedure on a manikin. These stains were captured by placing filtered papers across different locations, that surround the dental procedure area [15, 16, 18, 19, 26, 35, 37] and
- different sized particles using particle counters. Suspended particles that were generated while conducting a dental procedure on a manikin. [17–19, 29, 35, 36].

Studies were grouped according to the outcome that was explored as follows:

**Objective 1**. Change in contamination levels and spread pattern over distance from the dental procedure area **(*n*** = **18)**

### Description of studies

Eighteen studies investigated changes in contamination levels over distances from the dental procedure [15–18, 20, 21, 23, 24, 26–28, 30–34]. Details of the studies and the data on contamination levels are presented in Table 2. Whilst all investigated highspeed handpieces [20, 21, 24, 27, 28, 30–33], two also investigated micromotors [18, 23] and one looked at slow-speed handpieces [16]. Four studies reported instrument speed [16, 17, 24, 30]. To sample and measure contamination, nine studies used settle plates [20, 21, 24, 27, 28, 30–33], three studies [17, 18, 29, 36]. which presented evidence on four procedures used air sampling methods, seven used filter papers with fluorescein, [15–19, 26, 37], and one study [23], used both settle plates and air samplers.

**Table 2:** Contamination levels for different types of handpieces and outcome measures over distance from procedure site (*n* = 18).

Paper ID – Author, year	Speed (rpm)/ other info	Procedure Duration (Min)	Measure of contamination Any additional information			Distance from dental procedure site [exact distances where known]						
						0–30	31–60	61–99	100–199	200–299	300–399	≥400
**Type of handpiece - High-speed**												
Bently 1994 [21]	NI	2	**Settle plate (*n*** = 10)	CFU/plate Mean of 6 positions around patient		5	12.3	_	_	_	_	_
Manarte-Monteiro 2013 [27]	NI	60–240		CFU/plate Mean (SE)		_	16.60 (10.4)	_	_	13.60 (±6.9)	_	_
Purohit 2009 [30]	400,000	40		CFU/plate Mean (SE)		_	53.4 (4.5)	24.6 (5.0)	_	_	_	_
Samaranayake 1989 [32]	NI	5–15		CFU Mean (SE)		_	_	_	11.5 (4.9)	3.9 (0.8)	1.6 (0.3)	
Tag El-Din 1997 [33]	NI	5–15		CFU/plate Mean		_	_	25.1 (4.4)	_	20.4 (6.5)	_	_
Belting 1964 [20]	NI	1		Mycobacterium tuberculosis CFU (Total number)		3	17	_	10	_	_	_
Ionescu 2020 [24]	320,000	4		CFU/cm^2^ (mean)		0.5 [20 cm**]	0.5 [60 cm**] 0.42 [60 cm]	_	0.35 [120 cm] 0.28 [180 cm]	0.2 [240 cm]	0.18 [300 cm] 0.16 [360 cm]	_
Miller 1971 [28]	NI	0.3 (20 s)		CFU/ft^2^ Range		1000–10,000	100–1,000 [60 cm]	100–10	≤10		_	_
Rautemaa 2006 [31]	NI	40		Mean CFU/m^2^/h (position)		_	_	823 [<100 cm behind patient]	_	1120 [>150 cm in front of patient]	_	_
Hausler 1966 [23]	NI	NI		Colony count/ft^2^/min (mean)		>80 [26 cm]	<20 [51 cm]	<10* [76 cm]	_	_	_	_
Yamada 2011 [34]	NI	NI	**Air sampling (*n*** = **3)**	Blood presence positive % (ratio)	Crown Preparation	_	35% (9/26)	_	29% (6/21)	_	_	_
					Inlay preparation	_	70% (21/30)	_	48% (15/31)	_	_	_
Shahdad 2021 [17]	360,000	20		Particle concentration By size (mm^3^/m^3^)	Very small	0.02	_	_	<0.01*	_	_	_
					Small	>6*	_	_	<2*	_	_	_
					Medium	>0.01*	_	_	<0.005*	_	_	_
					Large	>0.1*	_	_	<0.05*	_	_	_
Hausler 1966 [23]	NI	NI		Colony count/ ft^2^/min Mean	>20* [26 cm]	<5* [51 cm]	<2* [76 cm]	_	_	_	_	
Han 2021 [16]	300,000	2 s	**Filter paper (*n*** = **6)**	Mean grey value (um) front of patient	80–120* [25–30 cm]	110* [60 cm]	_	_	_	_	_	
Holliday 2021 [15]	NI	NI		Fluorescein droplet count/ mm^2^ Mean (SE)	No suction	_	_	_	47,728 (39,888)	_	1987 (2888)	1205 (2489)
					Low volume suction	_	_	_	24,216 (38,750)	_	361 (478)	218 (434)
					Medium volume suction	_	_	_	22,234 (36,993)	_	370 (421)	207 (287)
Ahmed 2021 [37]	Two-hole handpiece	NI		Fluorescein droplets /mm^2^ (Mean value for 7 directions)	60	41	14	4	1	_	_	
Allison 2021(c) [35]	NI	10		RFU per surface area (mean)	Operator 9878	1514	_	581	166	_	_	
Allison 2021(a) [18]	NI	**10**		RFU per surface area (mean)	With Suction	671	20 [50 cm]	_	0.01 [100 cm] 0.04 [150 cm]	0 [200 cm]	0 [300 cm] 0 [350 cm]	0 [400 cm]
					No Suction	669	78 [50 cm]	_	1.47 [100 cm]1.5 [150 cm]	0 [200 cm]	0 [300 cm] 0 [350 cm]	0 [400 cm]
Llandro 2021 [26]	NI	10		Fluorescein droplets/ mm^2^ Mean (SE)	91.5 (127.8)	1.88 (5.7) [50 cm]	_	0.01 (0.05) [100 cm] 0 [150 cm]	0 [200 cm] 0 [250 cm]	0 [300 cm] 0 [350 cm]	0 [400 cm]	
				RFU per surface area Mean (SE)	2328 (2917)	1019 (3335) [50 cm]	_	251 (1230) [100 cm] 31 (153) [150 cm]	0 [200 cm] 9 (45) [250 cm]	59 (290) [300 cm] 0 [350 cm]	31 (151) [400 cm]	
**Type of handpiece–Micromotor**												
Ionescu 2021 [24]	Contra-angled with water 320,000	4	Settle plates	CFU/cm^2^ Mean	0.35 [20 cm**]	0.38 [60 cm**] 0.44 [60 cm]	_	0.4 [120 cm] 0.28 [180 cm]	0.18 [240 cm]	0.12 [300 cm] <0.02 [360 cm]	_	
Llandro 2021 [26]	_ (no water)	10	Filter paper	Fluorescein droplets per mm^2^ Count	0	0 [50 cm]	_	0 (100 cm) 0 (150 cm)	0 (200 cm) 0 (250 cm)	0 (300 cm) 0 (350 cm)	_	
				RFU per surface area Mean	0	0 [50 cm]	_	0 [100 cm] 0 [150 cm]	0 [200 cm] 0 [250 cm]	52 (±257) [300 cm] 0 [350 cm]	_	
**Type of handpiece–Slow-speed**												
Han 2021 [16]	1,200	2 s	Filter paper	Grey value (µm) mean Front of patient	>20* [25–34 cm]	~10* [60 cm]	_	_	_	_	_	

^*^Estimated from graphs as exact values not stated.

** Values obtained from samples taken on chair (all other results were room surfaces).

*NI* no information given, no measurement taken.

*CFU* colony forming units, *RFU* relative fluorescein units, *SE* standard error, *SD* standard deviation.

Studies investigated contamination at a variety of distances from 0 cm to 400 cm (see Table 2). To summarise the findings, the distances are presented as (0–30 cm, 31–60 cm, 61–99 cm, then 1–4 m, and over 4 m). Seventeen studies investigated contamination within an area of less than one metre from the procedure site. Eleven studies measured contamination within 1–2 m [15, 17, 18, 20, 24, 26, 28, 32, 34], and 10 studies explored it for distances beyond two metres [15, 17, 18, 24, 26, 27, 31–33].

### Contamination with high speed over distance

Ten studies used measures to detect contamination based on settle plates (all measuring colony forming units of bacteria), with one study [20] specifically looking at Mycobacterium tuberculosis. Six studies used filter paper placed to measure fluorescein added to the water lines. Three studies used air sampling.

### High-speed contamination over distances up to 60 cm

Nine studies measured the change between the immediate operating site and a distance of between 30 and 60 cm [15–18, 20, 21, 23, 26, 28]. Four used settle plates [20, 21, 23, 28], one also used air sampling [23], and five used filter paper-based collection methods [15–18, 26].

Findings for two studies [20, 21] using settle plates showed an increase in contamination between the site of the procedure and at distances of up to 1 m. Bently (1994) [21], noted the CFU/ plate increased from 5 to 12.3 at 0–30 cm and 31–60 cm, respectively, and Belting (1964) [20], found the number of colonies of Mycobacterium Tuberculosis increased from 3 to 17 at 0–30 cm and 31–60 cm from the procedure site respectively. Two studies found a decrease in contamination [23, 28].

Three studies [19, 23, 35] used air sampling. This was in addition to settle plates and the reduction in contamination levels found using air sampling and settle plates were similar.

Of the five studies that used filter paper to measure contamination, one study found no difference in contamination between 26 cm and 51 cm [16], but this study measured only in front of the patient and the remaining four studies [15, 17, 18, 26] found a reduction in contamination.

At distances up to 60 cm, there were mixed findings with most studies showing a reduction in contamination levels but a few showing an increase. The results indicate that in within 60 cm of the procedure, the position of the operator, nurse, and patient influence the measures, and there is not a straightforward reduction with distance.

### High speed contamination over distances from 60 cm

There were 16 studies [15, 17–20, 23, 24, 26–28, 30–34, 37], that investigated changes in contamination at distances over 60 cm for the high speed.

Nine studies used settle plates and a reduction in contamination levels over 60 cm was recorded in eight out of nine studies [20, 23, 24, 27, 28, 30, 32, 33], with one study [31], showing an increase in contamination at 2 m compared with less than 1 m however, the recording taken at less than 1 m was behind the patient whereas the one at 2 m was recorded from in front of the patient.

All three of the studies using air sampling methods [17, 23, 34] recorded a reduction in contamination over distances beyond 60 cm.

All five out of six studies [15, 16, 18, 19, 26, 37], looking at contamination using filter paper recording methods, found a reduction in contamination over distances beyond 60 cm. One study [26], found a drop in results to 0 relative fluorescein units [38] per surface area at 2 to 3 m but registered positive contamination beyond this distance.

Out of 16 studies investigating contamination levels with high speeds over distances greater than 60 cm, there was a reduction over distance found in 14 with two [26, 31] showing increases at some distances. For Rautemaa et al. (2006) [31] this could be explained by changes in positioning of the sampling relative to the operating site.

### Micromotor contamination over distances up to 60 cm (*n* = 1)

Only one study, using filter paper to detect contamination, looked at differences at distances less than 60 cm [24], and no contamination was found at distances up to 60 cm.

### Micromotor contamination over distances from 60 cm (*n* = 2)

Two studies looked at micromotors over distances greater than 60 cm [24, 26]. One used air sampling [24] and found a similar contamination level at 120 cm to that found at 60 cm but then a consistent reduction in contamination over distances up to 360 cm. Llandro et al. 2021 [26] used filter paper and found no detectable contamination using fluorescein droplets per mm^2^ over any distance. However, with the RFU per surface area [39], there was no detectable contamination at 1 m, 1.5 m, 2 m, 2.5 m, and 3.5 m but they found contamination at 3 m.

### Slow speed contamination over distances up to 60 cm (*n* = 1)

Only one study [16], investigated contamination over distance with slow-speed handpieces, and did this using a filter paper technique. There was a reduction in contamination between 25–30 cm and at 60 cm.

### Slow speed contamination over distances from 60 cm

No studies measured contamination by slow-speed handpieces over 60 cm.

**Objective 2**. Change in contamination levels over time following procedure **(*n*** = **7)**

Seven studies [15, 17, 22, 25, 32, 33], explored the changes in aerosol contamination level over time (Table 3). All reported on the use of high-speed handpieces, and none investigated micromotors or slow-speed handpieces. Four studies measured contamination levels before, during, and after the procedure.

**Table 3:** Contamination levels for different types of handpieces and outcome measures in relation to time before, during, and after procedure (*n* = 7)

Paper ID – Author, year	Handpiece speed (rpm)	Distance measures were taken	Procedure Duration (min)	Measure of contamination Any additional information		Before treatment	During treatment	Immediately following treatment	After treatment [Exact time] (min)						
									0–5	6–15	16–30	31–60	61–120	>120	NI
**Type of handpiece–high speed**															
Tag El-Din 1997 [33]	NI	100 cm	5–15	**Settle plate (*n*** = **3)**	CFU/plate	8.8 (3.65)	25.1 (4.43)	_	_	_	_	_	_	_	13.5 (4.45)
		200 cm			Mean (SE)	9.4 (1.58)	20.4 (6.50)	_	_	_	_	_	_	_	11.3 (1.95)
Larato 1966 [25]	NI	NI	1.5–5		CFU count/ ft^3^ /min Mean value of 12 teeth	3.4* (Operator & Nurse present)	75.6	_	_	_	8.5 [30]	_	_	_	_
Samaranayake 1989 [32]	NI	100 cm	5–15		CFU/plate Mean (SE)	1.5 (0.6)	11.5 (4.9)	_	_	2.1 (0.4) [10]	_	_	_	_	_
		200 cm				2.0 (0.5)	3.9 (0.8)	_	_	2.0 (0.5) [10]	_	_	_	_	_
		300 cm				1.4 (0.5)	1.6 (0.3)	_	_	1.5 (0.6) [10]	_	_	_	_	_
Grenier 1995 [22]	NI	NI	8	**Air sampling (*n*** = **2)**	CFU count/ m^3^ Mean (SE)	14 (4)	75 (22)	51 (22)	_	_	_	_	12 (4) [120]	9 (4) [240]	_
Shahdad 2021 [17]	360,000	NI	20		(Closed bay) Time (min) to return to pre-procedure levels (Median)	NI (implied from outcome)	_	_	7 (Max 8)	_	_	_	_	_	_
					(Open bay) Time (min) to return to pre-procedure levels (Median)	NI (implied from outcome)	_	_	5 (Max 17)	_	_	_	_	_	_
Ahmed 2021 [37]	NI	30 cm	30	**Filter paper (*n*** = **2)**	(Front of patient) Fluorescein droplet count/mm^2^ Mean (Standard Deviation)	NI	_	60.6 (11.9)	_	_	10.1 (5.7) [30]	_	_	_	_
		60 cm					_	40.8 (15.2)	_	_	0.5 (1.2) [30]	_	_	_	_
		90 cm					_	16.5 (10)	_	_	0 [30]	_	_	_	_
		120 cm					_	4.1 (5.1)	_	_	0 [30]	_	_	_	_
Holiday 2021 [15]	NI	NI	10			NI	_	_	2.2	0.2	0.0 [30]	NI	_	_	_
					Fluorescein RFU/mm^2^ Mean		_	_	69,315 [0–5]	2,488[10–15]	4,326 [20–25]	191 [30–40]	822 [60–70]	_	_

^*^Estimated from graphs as exact values not stated.

*NI* no information given, no measurement taken.

*CFU* colony forming units, *RFU* relative fluorescein units, *SE* standard error, *SD* standard deviation.

There were a wide range of post-procedure period times for which contamination levels were reported. These extended from immediately following treatment, to more than two hours after the procedure. One study (Tag El-Din) [33] did not specify when the measurement was taken. All studies showed a similar pattern with aerosol contamination considerably increasing after the procedure commenced, followed by a decrease when the procedure stopped. The decreasing levels over time varied amongst the studies. Samaranayake et al. 1989 [32], showed that contamination after 15 min was still higher than pre-procedure records when assessment was measured at 100 cm distance. Larato et al. (1966) [25], also found that contamination did not return to the original levels after 30 min of the procedure when it was measured at the operator and Nurse level. Tag El-Din [32] results displayed similar findings with no specific time gap after procedure was reported.

However, contamination returned to original levels when the assessments were carried out at further distances; such as at 200 cm and 300 cm distance from procedure location [32], or at a longer time from the procedure as Grenier et al. (1995) [22] has shown (Mean[SE]before 14[4]; after 120 min 12 [4]). Shahdad et al. (2021) [17], reported on the contamination change overtime differently and found it takes 7–8 min for the aerosol to be back to its original level for closed bay settings, and 5 (max 17) min for open bay clinics.

Out of the 18 studies, eight used the rotary handpiece for five minutes or less [15, 19, 20, 23, 24, 27, 28, 34], five studies [14, 17, 19, 25, 35] specified that the procedure times lasted exactly 10 min with three other studies [21, 31, 32] stating procedure times ranged from between five to 15 min. One study reported procedures were carried out for 20 min [16] and one study for 40 min [30]. The remaining four studies did not provide this information [22, 29, 33, 36].

### Contamination with high speed pre- and post-procedure (*n* = 5)

Five studies measured contamination levels pre- and post- procedure [17, 22, 25, 32, 33]. Two of the studies [17, 32], suggest that it would take around 10 min for the contamination levels to return to their pre-procedure levels in the environment of a closed bay dental setting. In the study by Larato et al. 1966 [25], the contamination level did not return to pre-procedural levels after 30 min. Grenier (1995) [22], measured the contamination level after a longer time (120 min) after the end of the procedure and noted that it returned to pre-procedural levels then but there is no information on contamination levels before this timepoint. Tag El-Din et al. (1997) [33], reported that the contamination level did not return to the pre-procedure levels. However, this study did not specify the time of the measurements after the procedure.

### Contamination with high speed during treatment and post-procedure (*n* = 4)

There were four studies that measured contamination during treatment (Table 3) [22, 25, 32, 33]. Three of them used settle plates to record this and Grenier (1995) [22] used air sampling. All reported a large increase in contamination during the procedure and all noted a reduction in the aerosol level after the procedure had been completed, although there were a wide variety of timepoints measured, making it difficult to detect any patterns.

**Objective 3**. Contamination levels with different types of handpieces **(*n*** = **6)**

Six studies compared two or more different types of handpieces [16, 19, 24, 26, 29, 36]. Four of them compared the high-speed handpiece to the electric micromotor [15, 24, 26, 29]. Two studies [16, 36], compared the amount of aerosol generated from the use of high-speed and slow-speed hand piece, and only one study that compared the three types against each other. The speeds of highspeed handpieces ranged from 200.000–400.000 rpm. For the micromotor, these were explored from 50.000–200.000 rpm. The aerosol generated of using the slow-speed handpiece was explored the instruments was rotating at a range of 1.200–15.00 rpm. One study [26], did not provide details on the speed (Table 4).

**Table 4:** Contamination levels of different types of handpieces where there are within study comparisons (*n* = 6).

Author, year	Procedure duration	Sampling technique	Outcome measure	Additional information	Type of handpiece				
					Air turbine/High-speed	Micromotor			Slow speed
Allison 2021(b) [19]	10 min			Handpiece Speed (rpm)	400,000	200,000 no water	120,000 no water	60,000 no water	
		Filter paper	Fluorescein tracer		>140.000	120.000–140.000	120.000–140.000	100.000–120.000	
		Air sampling	Suspended fluorescein-containing droplets counts	Distance					
				50 cm	400–600	>200	>200	>200	
				150 cm	~1000	>200	>200	>200	
				170 cm	200–400	>200	>200	>200	
Han 2021 [16]	2 s	Filter paper		Handpiece Speed (rpm)	300,000				1000
			Mean grey value (um) Median (Count)	Distance 120 cm	407 (32870)				200 (80)
Grzech-Lesniak 2021 [36]	1 min	Air sampling		Handpiece Speed (rpm)	200,000				1500
			Count of aerosol particles (Manikin site) Mean (±SD)	Saliva ejector High volume evacuator	235.2 (±18.80) 64.1 (±4.6)				183.0 (±8.1) 55.1 (±3.3)
Llandro 2021 [26]	10 min	Filter paper		Handpiece Speed (rpm)	No information	No information on speed (No-water)/ortho procedure			
			Count of fluorescein droplets/mm^2^ Mean (±SD)		5.21 (±5.82)	0.42 (±1.26)			
			Relative fluorescence units per surface area Mean (±SD)		1723 (±3280)	444 (±1.34 4)			
Ionescu 2020 [24]	4 min	Settle plates		Handpiece Speed (rpm)	320,000	Contra-angled handpiece with water 50,000			
			Colony-forming units (CFU) per square centimeter (CFU/cm^**2**^)	Distance 20 cm 60 cm 60 cm 120 cm 180 cm 240 cm 300 cm 360 cm	0.5 0.5 0.42 0.35 0.28 0.2 0.18 0.16	0.35 0.38 0.44 0.4 0.28 0.18 0.12 <0.02			
Nulty 2020 [29]	2 min	Air sampling		Handpiece Speed (rpm)	No information	Micromotor handpiece with water			No information
			Maximum particulate counts for different particle sizes during each procedure (μg/m^3^) Mean	Particle sizes* PM1 HVE PM1 No HVE PM2.5 HVE PM2.5 No HVE PM10 HVE PM10 No HVE	6 6 10 9 24 11	5 4 11 6 23 9			4 4 10 5 16 7

^*^PM1 (particle size <1 μm), PM2.5 (particle size 1–2.5 μm) and PM10 (particle size 2.5–10 μm).

### Contamination of highspeed compared to micromotors

Of the four studies [15, 24, 26, 29] where within study comparisons could be made between high speeds and micromotors, two [19, 26] found reductions in contamination for micromotors and two studies [24, 29], found little difference. In the study by Ionescu et al. [24] (using settle plates and CFUs), the values were slightly lower for the contra-angled handpiece apart from at 60 cm and 120 cm where they were slightly higher. The maximum distance of tracer detection was 360 cm for high-speed air turbine, 300 cm for the micromotor contra-angle handpiece. Nulty et al. (2020*)* [29], (Using air sampling) reported with particle sizes 1–2.5 μm and HVE that the maximum particulate count increased slightly. However, there were no speeds given for the handpieces.

Two studies [19, 26] stated that no water spray was used with the micromotors whereas water spray was used by Ionescu et al. (2020) [23] and Nulty et al. (2020) [28] where less, and at some distances no reduction, in contamination levels were found.

#### Contamination with high speed and micromotors compared to slow speed handpieces

There were three studies that investigated slow speed handpiece contamination levels relative to other handpieces. Two compared slow with high speeds [16, 36], with less contamination associated with use of the slow speed compared to high-speed handpieces. One study [29], compared slow speed with both high speed and micromotors using air sampling and recording contamination for different particle sizes. They reported reduced contamination for slow speed compared to high speed for all particle sizes apart from Particle Matter (PM) PM size 2.5 mm with High Volume Evacuation (HVE) (this was similar to micromotor compared to high-speed findings). There was also no difference between slow speed and micromotor for PM size 1 where no HVE was used.

### Statistical data analyses

There was a great deal of heterogeneity between the outcomes, the outcome measures, and the parameters they were measured against (timepoints and distances). In addition, there were other factors related to the procedures that differed between each study (make, model, and rotation speed of the handpieces). The environments varied and different studies used a variety of adjuncts such as high-volume aspirators, saliva ejectors, and the amount of water flow through the handpieces. These variations meant it was not possible to carry out any statistical analyses or meta-analysis to investigate the findings further.

### Sampling sensitivity analysis

Sensitivity of the aerosol sampling techniques was assessed according to the type of the contamination generated.

### Sensitivity groupings of sampling/culturing techniques (*n* = 23)

For the microbial contamination, Table 5 shows ten out of 11 studies used blood agar as a medium to incubate bacteria. The incubation environment was not explicitly described in most of the studies. For other methods that were used to assess contamination such as fluorescein stain or particle counts analysis, six were deemed to be of a high sensitivity, one was low and two were moderate. There was one study that reviewers could not reach a decision as no details were mentioned. Table 7 shows the sensitivity analysis of the single study [34] that used blood which deemed to be moderate in sensitivity (Tables 5–7).

**Table 5:** Sensitivity measure of bacterial detection (*n* = 12).

	Study	Sampling method	Blood agar used	Incubation environment	Incubation duration	Sensitivity assessment
1	Belting 1964 [20]	Settle plates	Yes	Not stated	48 h	Low
2	Grenier 1995 [22]	Air sampler	Yes	Anaerobic	7 days	High
3	Hausler 1966 [23]	Both	No	Not stated	Not stated	Low
4	Manarte-Monteiro 2013 [27]	Settle plates	Yes	Not stated	48 h	Low
5	Purohit, 2010 [30]	Settle plates	Yes	Not stated	24 h	Low
6	Rautemaa 2006 [31]	Settle plates	Yes	Not stated	48 h	Low
7	Samaranayake 1989 [32]	Settle plates	Yes	Aerobic	48 h	Low
8	Miller 1971 [28]	Settle plates	Yes	Not stated	48 h	Low
9	Belting 1964 [21]	Settle plates	Yes	Aerobic	15 weeks	High
10	Tag El-Din 1997 [33]	Settle plates	Yes	Aerobic	48 h	low
11	Ionescu 2020 [24]	Settle plates	Yes	Anaerobic	48 h	Low

**Table 6:** Sensitivity measure of studies used other methods to measure contamination (non-microbial/ non-blood based) (*n* = 10).

	Study	Visible inspection alone	Visible inspection with enhancers	Highly sensitive equipment used e.g., SEM.	Overall sensitivity assessment
1	Bentley 1994 [20]	Yes	–	–	Low
2	Ahmed 2021 [34]	–	Yes	–	Moderate
3	Allison 2021(a) [18]	–	Yes	–	Moderate
4	Allison 2021(b) [19]	–	Yes	Yes	High
5	Allison 2021(c) [35]	–		Yes	High
6	Grzech-Lesniak 2021 [36]	–	–	Yes	Unscored as sensitivity and equipment not specified
7	Han 2021 [16]	–	–	Yes	High
8	Nulty 2020 [29]	–	–	Yes	High
9	Holliday 2021 [15]	–	–	Yes	High
10	Shahdad 2021 [17]	–	–	Yes	High

**Table 7:** Sensitivity measure of studies used blood to measure contamination (non-microbial / non-blood based) (*n* = 1).

Included studies (First author year)	Visible inspection alone	Visible inspection with enhancers	Highly sensitive equipment used e.g., PCR	Overall sensitivity assessment
Yamada 2011 [34]	–	Yes	–	Moderate

#### Study quality assessment (*n* = 23)

Across all 23 studies, quality assessments were conducted by assessing the studies against seven key domains. Table 8 shows that none of the studies included in the review conducted a sample size assessment a priori. The second lowest quality domain was whether the studies included a control to compare their results against. Only 12 studies reported that they included controls in their investigations. Twelve studies were not microbial measurements; hence this domain did not apply to them. The highest quality domain was around conflict of interests with no study reporting any potential conflict of interest due to external funding or affiliations. For more details, please see the traffic light table and the summary below:

**Table 8:**
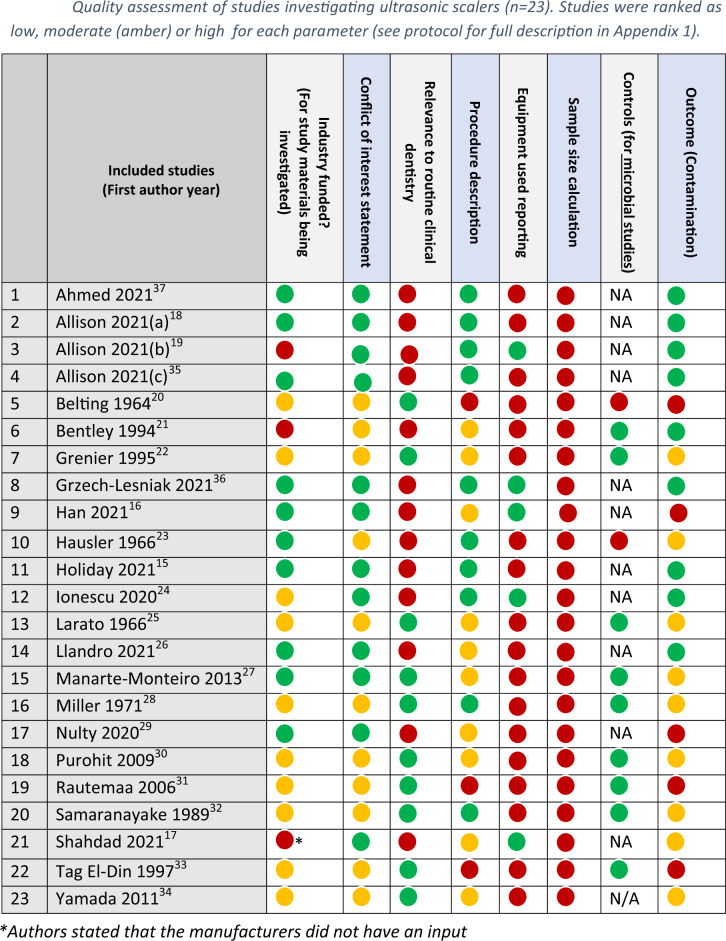
Quality assessment of studies investigating ultrasonic scalers (*n* = 23). Studies were ranked as low, moderate (amber) or high for each parameter (see protocol for full description in Appendix 1).

## Discussion

This systematic review included 23 studies and it updates some of the information in a broader systematic review carried out over a year ago [7]. It included twelve studies from the previous review and 11 studies published since then with information on the use of dental handpieces during restorative and restorative-like procedures [15–19, 24, 26, 29, 36].

This review is based on what might now be considered a historic or outdated perception but is nevertheless still a commonly held assumption; that aerosols contain generated particles of less than 5 μm in diameter. However, this cut-off point is not necessarily based on solid evidence as thoroughly explained by Randall et al. in 2021 [1]. The authors assert that “In particular, current recommendations have been based on four tenets: (i) respiratory disease transmission routes can be viewed mostly in a binary manner of ‘droplets’ versus ‘aerosols’; (ii) this dichotomy depends on droplet size alone; (iii) the cut-off size between these routes of transmission is 5 µm; and (iv) there is a dichotomy in the distance at which transmission by each route is relevant. Yet, a relationship between these assertions is not supported by current scientific knowledge.” Hence, a less absolute position should be taken in future and more multi-disciplinary research efforts are needed to have a better understanding of aerosol characteristics and its impact on disease transmission within the field of Dentistry.

Overall findings for the outcomes investigated were: (1) distance; reduction in aerosol and splatter levels at different distances seemed to be influenced by the direction of measurement and the methods used to generate and detect spread and settle (2) time; there was general reduction in the levels of contamination over time following the procedure but again, there was a mixed picture that seemed to depend on investigative methods which meant that no firm conclusions could be drawn from the research specifically on the time taken for levels to return to pre-procedural levels; and (3) different handpieces: using slow-speed handpiece generated less contamination than using the faster types.

However, there were less conclusive findings from four studies [19, 23, 25, 28] investigating contamination generated from micro-motor handpieces, with the procedures using water showing less reduction compared to high-speed handpieces. The reduction in contamination detected when micromotor and slow speed handpieces were used seemed to be related to reduced rpm but the use of water in some micromotor handpieces appeared to bring this back to levels comparable to high-speed handpieces.

For distance, all 18 studies investigating changes in contamination examined high-speed handpieces. Two of these also included micromotor handpieces and one included slow-speed handpieces. They found that generally, there was a reduction in contamination at distances greater than 60 cm with 14 studies reporting consistent reductions as distance from the procedure increased and two [26, 31] showing some increases in contamination levels for some distances. This finding, was explained by the authors of those investigations as being due to limitations in sample collection techniques [26, 25]; dry aerosol contamination was detected when photographic analysis was used, but debris was not absorbed, and hence not captured when the filter papers were analysed. For the nine studies looking at distances less than 60 cm there was a mixed picture. Six studies found a reduction in contamination [17–19, 23, 26, 28]; one found no change [16]; and two [20, 21] showed an increase between the procedure site and the distances up to 60 cm. For the two studies showing increases, this may have been related to the position that the measurement was taken, in relation to the procedure/ and staff (operator/nurse), or the location in relation to the patient (i.e., behind or in front), but there was not a consistent, incremental reduction in contamination over short distances. Five studies demonstrated how the position of the measurement being taken in relation to the patient influenced contamination level measurements through heatmaps [18, 19, 21, 24, 26].

Investigation of contamination levels over time following procedures involved seven studies which explored changes in aerosol contamination levels, but these only investigated high-speed handpieces. Five looked at contamination levels pre- and post-procedure with two reporting return to pre-procedure levels taking around 10 min, one finding this to take 30 min [25], and the other [22], reporting it as two hours. Shahdad et al. (2021) [17], measured fallow time directly and has highlighted the vital role that ventilation plays in clearing the contamination level after the procedure as they found the clearing (i.e fallow) time can be up to 90 min if no ventilation is employed. This reinforces the critical role of ventilation in reducing the time taken for contamination levels from AGPs to return to pre-procedural levels.

When comparing different types of handpieces, use of a slow-speed handpiece consistently showed lower contamination levels than micromotors (one study) and high speeds (three studies). Micromotors reduced the contamination compared to high speeds. This seems likely to be related to speed of the handpiece but there was less difference between contamination levels when micromotors were used with water and sometimes the contamination measured was similar or even slightly higher (although this could be within margins of error).

Synthesising the data for this review was significantly hampered by three factors: firstly, diverse methodological heterogeneity, secondly missing information in some reports (notably air exchange in environments) and finally, a lack of understanding around the implications for different types of methodology which can be explained as follows. Ten studies used bacteria-based measurements for assessing the changes of aerosol contamination levels. However, bacterial aerosol contamination is associated with larger particles of 2 µm or more because of the relatively large mass of bacteria. Hence these studies may have assessed the extent of splatter but may not have addressed contamination from aerosol generated from dental procedures and may have less direct relevance to the spread and settle of aerosolised viral material. This is especially true as dental aerosol particle size generated from dental procedures has been shown to be around 0.05–0.07 µm [40]. These nanosized particles behave differently to larger ones, with one lab-based study [41] suggesting that they can stay suspended in the air for hours and can travel long distances. Furthermore, a study by Leung et al. (2020) [42], has detected coronavirus RNA in the aerosol within this nano-size aerosol contamination. SARS-CoV-2 virions are around 0.08 µm in diameter and thus could easily be spread via aerosol [43].

Six studies, most of which were recently published in response to this pandemic, used fluorescein dye to measure contamination by introducing it into water systems or dropping it into the mouth, mimicking saliva. The spread and settle of the fluorescein were captured through use of filter papers placed around the operatory. Visual inspection or photographic image analysis complemented by spectrofluorometric analysis were used to measure the levels of fluorescein. Larger splatters are likely to indicate the detection of particles rather than microscale aerosol. Only one study [16], used a fluorescein-detecting microscope to count the small aerosol spots across a distance of 120 cm from the procedure site. The authors displayed the results diagrammatically showing that different sizes of particles classed as very small [0.08–0.26 µm], small [0.27–0.90 µm], medium [0.91–2.70 µm], and large [2.71–10 µm] generated different particle concentrations (measured as mm^3^/m^3^). At 120 cm, small size particles (0.27–0.90 µm) showed by far the greatest concentration. However, there are complications in interpreting these data as the methodology states that the filter papers used have a maximum retention size of 2 µm and also fluorescein was placed in the water reservoir which, as demonstrated by the differences seen by Llandro (2021) [26], and Allison (2020) [18], may lead to overestimation of the amount of particulate matter in the air.

Some additional limitations that present difficulties interpreting the level and pattern of aerosol contamination were explained by Allison et al. 2021 [19]. Fluorescein particles can be deformed when they settle on the cotton-based filter papers, as the liquid stain expands through the paper and increases in size. If the area is heavily contaminated, then the fluorescein stain spots join leading to potential inaccurate results (if numbers of spots are counted). Other studies used particle counting to assess both the amount and particle size generated from dental procedures. However, two studies [19, 36], combined all particle sizes. One other study [29], only identified particle sizes of 1 µm or more. However, as discussed previously, this cut-off size may not be sensitive enough to detect aerosol spread. One study [17], provided some evidence on very small (0.08 µm) particles level after the use of rotary handpiece instruments. The authors found a general reduction in contamination levels. However, the concentration of the “very small” particles travelling to the adjacent and opposite bays (at a distance of 170 cm), did not change even after 30 min following procedure completion. This finding strongly indicates the need to consider the impact of different particle sizes on the AGPs-related contamination levels when designing research.

### Limitations

The systematic review has several limitations. The variety of methodologies employed by the primary studies meant it was not possible to carry out statistical meta-analysis of any data. Eleven out of 12 studies published before 2020 investigated dental treatments which were carried out on patients but only one of the 12 studies carried out since 2020 [24], assessed the bioaerosol (specifically microbial) generated from conducting a procedure on a patient (Table 1). The COVID-19 pandemic has hampered such investigations, but some methodologies could be adapted to mimic clinical environments by using mannequins and in simulated clinics. However, one of the implications of this is the difference between measuring contamination from solution spread when the fluorescein dye being measured is in the fluid reservoir as opposed to saliva [15, 16, 18, 19, 26, 35, 37]. One group who published five of recent studies, used slightly different methodologies to investigate different aspects, as noted in one of their most recent papers [35], “The contamination readings obtained in the present study by using fluorescein in the mouth of the mannequin were significantly lower for the positive control condition (anterior crown preparation with suction) than we have previously reported using fluorescein in the irrigation reservoirs of dental instruments [35]. This is perhaps unsurprising but demonstrates that only a small proportion of the settled aerosol and splatter produced by dental procedures is likely to be made up of saliva (and/or blood). This dilution effect should be the subject of further study but indicates that this model is likely to be more biologically and clinically relevant”. This finding, together with indications that position around the patient influences contamination levels and the lack of specific aerosol measurement, underpin the need for the profession to come together to standardise methods for assessing aerosol and splatter generation and agree tools for assessing these to allow more efficient research workflows and synthesising/ comparison of data under different circumstances.

It is worth highlighting that there is a significant limitation that they are only directly relevant to the particular setting in which they were carried out, and external validity is difficult to rely on. This is because of the variation in room size, airflows, directionality of aerosol plumes, and ventilation rates in the setting etc.

Since the start of the Covid-19 pandemic, the delivery of clinical dentistry has been severely restricted by fears of, and measures taken to prevent, airborne transmission of the SARS CoV-2 virus. Hence, there is a need to make most of the data generated from this period to learn more on what can be done to make dentistry more sustainable in the face of the similar challenges in the future. The concept of the AGP was prominent in restricting and modifying dental care according to the amount the aerosol generated from these procedures. However, transmission risk is a function of a number of factors, including; viral load, humidity level, proximity, duration of exposure, and quality of ventilation. Relying on cataloguing a list of AGPs/non-AGPs is too simplistic an approach [43]. Therefore, we need look beyond a binary AGP/ non-AGP concept and consider how dentistry can be future-proofed to maintain services in the case of other future respiratory-borne viral threat. This seems to be vital considering the current pandemic situation and the expected emerging variants that may be more transmissible. More focus on dental setting ventilation, and the layout of premises including open plan settings is as important a focus as the dental procedures themselves [44]. Additionally, risk of transmission in a vibrant setting like the dental practices is perceived differently by patients and dental staff and this issue needs consideration when exploring the risk of transmission of infections.

## Conclusion

Although they should be interpreted with caution because of the limited quality of the data, the following conclusions can be drawn from this review of the literature on the behaviour of dental aerosols generated from dental handpieces:

- There are widely varying methodologies and different outcomes, which makes synthesising the data difficult and it was not possible to carry out any meta-analyses of the data.
- By using proxy measures such as bacterial contamination (which are relatively large) to detect contamination from AGPs, some studies may have measured droplet and splatter spread and settle but may have missed detecting aerosol.
- Placing tracer materials in water reservoirs does not accurately reflect the amount of spread from pathogens in oral fluids (saliva) and likely overestimates the amount in aerosol, droplets, and splatter.
- Distance: Dental aerosol contamination levels reduce the further away from the procedure they are measured. To a small extent, this reduction can be influenced by the direction of measurement from patient as well as the study design.
- Time: Contamination levels decrease as time passes following the end of the procedure. However, the studies did not find a consistent length of time by which contamination levels returned to pre-procedural levels. This was likely to be due to the varying methodologies and measurement techniques.
- The use of different handpieces: The studies consistently found that higher levels of aerosols were generated with handpieces using higher rpm and water spray. The highest aerosol levels were with use of high-speed handpieces, less aerosol was generated with use of micro-motor handpieces (although increased when water spray was used) and in turn these were even lower when slow-speed handpieces were used.

## Supplementary information

Supplementary Information

## References

[1] Harrel SK, Molinari J (2004). Aerosols and splatter in dentistry: a brief review of the literature and infection control implications. J Am Dent Assoc.

[2] Leggat PA, Kedjarune U (2001). Bacterial aerosols in the dental clinic: a review. Int Dent J.

[3] Gallagher JE, Johnson I, Verbeek JH (2020). Relevance and paucity of evidence: a dental perspective on personal protective equipment during the COVID-19 pandemic. Br Dent J.

[4] Banakar M, Bagheri Lankarani K, Jafarpour D (2020). COVID-19 transmission risk and protective protocols in dentistry: a systematic review. BMC Oral Health.

[5] Scottish Dental Clinical Effectiveness Programme [SDCEP]. Rapid Review of Aerosol Generating Procedures in Dentistry, https://www.sdcep.org.uk/published-guidance/covid-19-practice-recovery/rapid-review-of-agps/ (2021, accessed February 2022).

[6] Innes N, Johnson IG, Al-Yaseen W (2021). A systematic review of droplet and aerosol generation in dentistry. J Dent.

[7] Gallagher JE, Johnson IG KCS (2020). A systematic review of contamination (aerosol, splatter and droplet generation) associated with oral surgery and its relevance to COVID-19. BDJ Open.

[8] Johnson IG, Jones RJ, Gallagher JE (2021). Dental periodontal procedures: a systematic review of contamination (splatter, droplets and aerosol) in relation to COVID-19. BDJ Open.

[9] Nicola I, Ilona J, Waraf A-y, Rebecca H, Rhiannon Jo, Sukriti KC, Scott M, Mark R, William W, Jennifer G. A systematic review of aerosol, spatter and droplet generation in dentistry. PROSPERO 2020 CRD42020193058 Available from: https://www.crd.york.ac.uk/prospero/display_record.php?ID=CRD42020193058

[10] Page MJ, McKenzie JE, Bossuyt PM (2021). The PRISMA 2020 statement: an updated guideline for reporting systematic reviews. BMJ.

[11] Standard Protocol Items: Recommendations for Interventional Trials (SPIRIT). Guidance for Clinical Trial Protocols, https://www.spirit-statement.org/, (2019, 6 September 2020).

[12] Ouzzani M, Hammady H, Fedorowicz Z (2016). Rayyan—a web and mobile app for systematic reviews. Syst Rev.

[13] Thompson S, Ekelund U, Jebb S (2011). A proposed method of bias adjustment for meta-analyses of published observational studies. Int J Epidemiol.

[14] Briere J-B, Bowrin K, Taieb V (2018). Meta-analyses using real-world data to generate clinical and epidemiological evidence: a systematic literature review of existing recommendations. Curr Med Res Opin.

[15] Holliday R, Allison JR, Currie CC (2021). Evaluating contaminated dental aerosol and splatter in an open plan clinic environment: Implications for the COVID-19 pandemic. J Dent.

[16] Han P, Li H, Walsh LJ (2021). Splatters and aerosols contamination in dental aerosol generating procedures. Appl Sci.

[17] Shahdad S, Hindocha A, Patel T, et al. Fallow time determination in dentistry using aerosol measurement in mechanically and non-mechanically ventilated environments. Br Dent. J. 2021;34:1–8. 10.1038/s41415-021-3369-1 PMC8390043 34446842

[18] Allison JR, Currie CC, Edwards DC (2021). Evaluating aerosol and splatter following dental procedures: Addressing new challenges for oral health care and rehabilitation. J. Oral. Rehabil.

[19] Allison JR, Edwards DC, Bowes C (2021). The effect of high-speed dental handpiece coolant delivery and design on aerosol and droplet production. J Dent.

[20] Belting CM, Haberfelde GC, Juhl LK (1964). Spread of organisms from dental air rotor. J Am Dent Assoc.

[21] Bentley CD, Burkhart NW, Crawford JJ (1994). Evaluating Spatter And Aerosol Contamination During Dental Procedures. J Am Dent Assoc.

[22] Grenier D (1995). Quantitative analysis of bacterial aerosols in two different dental clinic environments. Appl Environ Microbiol.

[23] Hausler WJ, Madden RM (1966). Microbiologic comparison of dental handpieces 2. Aerosol decay and dispersion. J Dent Res.

[24] Ionescu AC, Cagetti MG, Ferracane JL (2020). Topographic aspects of airborne contamination caused by the use of dental handpieces in the operative environment. J Am Dent Assoc (1939).

[25] Larato DC, Ruskin PF, Martin A (1966). Effect of a dental air turbine drill on the bacterial counts in air. J Prosthet Dent.

[26] Llandro H, Allison JR, Currie CC (2021). Evaluating splatter and settled aerosol during orthodontic debonding: implications for the COVID-19 pandemic. Br Dent J.

[27] Manarte-Monteiro P, Carvalho A, Pina C (2013). Air quality assessment during dental practice: Aerosols bacterial counts in an universitary clinic. Rev Portuguesa de. Estomatologia e Cir Maxilo-facial.

[28] Miller RL, Micik RE, Abel C (1971). Studies on dental aerobiology: II. Microbial splatter discharged from the oral cavity of dental patients. J Dent Res.

[29] Nulty A, Lefkaditis C, Zachrisson P, et al. A clinical study measuring dental aerosols with and without a high-volume extraction device. Br Dent J. 2020; 1–8. 10.1038/s41415-020-2274-3. 2020/11/14. 10.1038/s41415-020-2274-3 PMC7658616 33184481

[30] Purohit B, Priya H, Acharya S (2009). Efficacy of pre-procedural rinsing in reducing aerosol contamination during dental procedures. J Infect Prev.

[31] Rautemaa R, Nordberg A, Wuolijoki-Saaristo K (2006). Bacterial aerosols in dental practice—a potential hospital infection problem?. J Hosp Infect.

[32] Samaranayake LP, Reid J, Evans D (1989). The efficacy of rubber dam isolation in reducing atmospheric bacterial contamination. ASDC J Dent Child.

[33] Tag El Din AM, Ghoname NAH (1997). Efficacy of rubber dam isolation as an infection control procedure in paediatric dentistry. La Rev de Sante de la Mediterr Orient.

[34] Yamada H, Ishihama K, Yasuda K (2011). Aerial dispersal of blood-contaminated aerosols during dental procedures. Quintessence Int.

[35] Allison JR, Dowson C, Pickering K (2021). Local exhaust ventilation to control dental aerosols and droplets. J Dent Res..

[36] Grzech-Leśniak K, Matys J (2021). The effect of Er:YAG lasers on the reduction of aerosol formation for dental workers. Materials.

[37] Ahmed MA, Jouhar R. Dissemination of aerosol and splatter in clinical environment during cavity preparation: an in vitro study. Int J Environ Res Public Health. 2021;18: 10.3390/ijerph18073773. 10.3390/ijerph18073773 PMC8038515 33916609

[38] Rodrigues R, Fernandes MH, Bessa Monteiro A (2019). Are there any solutions for improving the cleft area hygiene in patients with cleft lip and palate? A systematic review. Int J Dent Hyg.

[39] Randall K, Ewing ET, Marr L, Jimenez J, Bourouiba L (2021). How did we get here: what are droplets and aerosols and how far do they go? A historical perspective on the transmission of respiratory infectious diseases. Interface Focus.

[40] Polednik B (2021). Exposure of staff to aerosols and bioaerosols in a dental office. Build Environ.

[41] van Doremalen N, Bushmaker T, Morris DH (2020). Aerosol and surface stability of SARS-CoV-2 as compared with SARS-CoV-1. N Engl J Med.

[42] Leung NHL, Chu DKW, Shiu EYC (2020). Respiratory virus shedding in exhaled breath and efficacy of face masks. Nat Med.

[43] Lee BU, Minimum sizes of respiratory particles carrying SARS-CoV-2 and the possibility of aerosol generation. Int J Environ Res Public Health 2020; 17. 10.3390/ijerph17196960. 2020/09/27 10.3390/ijerph17196960 PMC7579175 32977575

[44] Hamilton F, Arnold D, Bzdek BR (2021). Aerosol generating procedures: are they of relevance for transmission of SARS-CoV-2?. Lancet Respir Med.

