# Supplementary material for: Aerosol and splatter generation with rotary handpieces used in restorative and orthodontic dentistry: a systematic review
Source: BDJ Open. 2022 Sep 6;8:26. doi: 10.1038/s41405-022-00118-4 (PMC9447949; doi:10.1038/s41405-022-00118-4)
Supplement: Supplementary file 1 — Supplementary Information [file 41405_2022_118_MOESM1_ESM.pdf]

Extracted data

Unique Study ID	Study author and reference	If the duration of the procedure was stated, please enter it here	If applicable, what was the main equipment used in performing the procedure? (e.g.rotary handpiece, ultrasonic scaler)	If stated and considered relevant, what was the working mechanism and/or the rotation/vibration speed of the main equipment used	Is the equipment used in performing the dental procedure relevant to today's practice (Please choose from the dropdown list Yes, No, N/A)	Was any additional equipments used as a standard measure e.g. rubber dam, suction) (Please choose from the dropdown list Yes, No, N/A)	If yes, Please specify in here	Person e.g. dental operator, dental nurse/ assistant, patient (choose from dropdown list; Yes, No, N/A)	If Yes please specify who and which parts of body
**99**	**Ahmed 2021**	3 minutes	Two-hole AA87handpiece (PANA MAX, NSK, Tochigi, Japan)<br>- four-hole handpiece (KAVO, Biberach, Germany).	high-speed handpiece	Yes	Yes	High volume suction (DSEplus Type 5193, KAVO,Warthausen, Germany)	Yes	operator chest, head, forearms, upper leg, and inside facemask. assistant: chest, head, forearms, upper leg, and inside facemask.
100	**Allison 2021(a)**	High Speed Air Turbine = 10mins 3-in-1 Spray = 30 seconds (replicate washing acid etchant)	Air turbine - Synea TA-98, W&H (UK) LTD and 3-in-1 Spray = Model not stated	high- speed handpiece. 3-in1 = air and water 140.6mL / min	yes	Yes	Suction for 3-in-1 measure at 6.3L of water per minute. Measured with and without suction for high speed	Yes - simulated body of Mannequin and Operator and Assistant	Above mannequin mouth. 4 papers on body of mannequin x2 40cm from hub and x2 80cm.<br><br>**Assistant** and Operator -upper mid forearm, upper chest, upper mid-thigh, Full-face visor and vertex of the head.

									Additionally for anterior crown prep with suction plus assistant, 3 filter papers were placed on the mask (beneath a full-face visor).
120	**Allison 2021(b)**	10 minutes	A speed-increasing handpiece (no water) High-speed	Highspeed: Approx: 400.000rpm<br><br>Micromotor: 60.000rpm; 120.000rpm; 200.000rpm	yes	yes	suction (flow rate 105l/min)	Yes - simulated body of Mannequin and Operator and Assistant	Above mannequin mouth. 4 papers on body of mannequin x2 40cm from hub and x2 80cm. Assistant and Operator -upper mid forearm, upper chest, upper mid-thigh, Full-face visor and vertex of the head. Additionally for anterior crown prep with suction plus assistant, 3 filter papers were placed on the mask (beneath a full-face visor).
127	**Allison 2021(c)**	10 minutes	High-speed air-turbine (Synea TA-98, W&H (UK) Ltd.; St Albans, UK).	no	yes	yes	medium volume suction (dental suction with an 8.3 mm internal diameter suction tip at a flow rate of 133 L/min)	Yes	Active air sampling: Operator: the left chest pocket
93	**Belting 1964**	1 minute	Air rotor	The dental air rotor (Borden Airotor, Ritter Co., Inc., Rochester, N. Y.) with no. 171 bur attached operating at an air flow pressure of 30 psi using 30 drops of oil and 18ml water per minute	YES?	Yes	air rotor was used with and without water	No	N/A

8	**Bentley 1994**	Experiment 1) 2 minutes (spatter) Experiment 2a) 30 minutes (aerosol with high speed Experiment 2b))30 minutes (aerosol with ultrasonic)	1) high speed handpiece 2 high speed handpiece	Not stated	Yes	Yes	1) High volume aspirator with high speed handpiece tooth preparation 2a) high volume aspirator with high speed handpiece 2b) saliva ejector with ultrasonic scaler	Yes	Operator: headcaps, masks and gowns. Dental Assistant: headcaps, masks and gowns. Patient: chest
29	**Grenier 1995**	High-speed drilling= 8 minutes	High speed handpiece	not stated	Yes	Yes	rubber dam	No	N/A
122	**Grzech-Lesniak 2021**	NA	highspeed handpiece W&H Synea TA-98LC (W&H, Bürmoos, Austria) low-speed handpiece W&H Synea TA-98LC (W&H, Bürmoos, Austria)	200,000rpm 15,000rpm	Yes	Yes	1) saliva ejector (SE) EM15 (Monoart® Euronda, Vicenza, Italy) and (2) high-volume evacuator (HVE) EM19 EVO (Monoart® Euronda, Vicenza, Italy). Evacuators were placed at the level of the tooth around 2 cm from its buccal side.	Yes	Operatore mouth: 2 cm away Assistant: Mouth : 2cm away Patient (simulated): mputh
107	**Han 2021**	Low speed: 5 min Highspeed: 5s triple syringe: 15s	Air turbine handpiece ((TiMax, NSK, Kanuma, Japan) low-speed air turbine handpiece (W&H implantMED™, Bürmoos, Austria)	300,000 rpm 1200 rpm	Yes	None	N/A	Yes	Operator: 20cm representing dentist position Assisstant: 29 cm representing the Assistant position

			Triple syringe						
**36**	**Hausler 1966**	not clear	high speed handpiece and air turbine	2.000 rpm	No	No	This is an old study, here is relevance bt the handpieces etc may not be the same	No	NA
**108**	**Holliday 2021**	10 minutes	Highspeed Hand piece.	not stated	Yes	Dental suction at two flow rates: low volume suction, 40 L/min of air; medium volume suction, 159 L/min of air.		No	na
**109**	**Ionescu 2020**	4 minutes	* Highspeed: Air turbine handpiece (Bora Led, Bien-Air Dental) equipped with a cylindrical diamond bur (835KR.314.016, Komet Italia Srl).<br><br>**Slow-speed: contra-angle handpiece (CA 1:1, Bien-Air) with a round tungsten carbide bur (H1SM.204.020, Komet) inside the already prepared cavity.	320,000 rpm.<br>50.000 rpm	Yes	N/A	N/A	Yes	Patient: 14 sites on the dental chair **Assisstant**: 1 on the assistant pad

91	**Larato 1966**	1.5-5 mins.	air turbine drill	N/A	YES?	Yes	Unit aspirating system	N/A	N/A
113	**Llandro 2021**	10 minutes	* speed-increasing dental handpiece driven by a dental air motor at full speed (with no water coolant)		Yes	yes	Large bore dental suction	Yes	Operator: forearms, chest, upper leg and head, their masks and full-face visor. assisstant: forearms, chest, upper leg and head, their masks and full-face visor.
50	**Manarte-Monteiro 2013**	1-4 hours	Manual (endodontic) + high-speed handpieces (turbine) (for (endodontic & restorative)	rotary action, with water-cooling;	Yes	Yes	Rubber dam	NA	NA
90	**Miller 1971**	20 seconds	Air turbine Handspeed+ 557 bur (multible procedures). Rubber cup and pumice (periodontics).	N/A	YES?	Yes	High velocity suction	No	N/A
115	**Nulty 2020**	1 Minutes	Electric Micromotor: " Micromotor high-speed handpiece with water"Mircomotor Air turbine: HIGH SPEED WITH WATER Low speed with water Three-in-one		Yes	yes	saliva ejector	no	N/A
60	**Purohit 2009**	N/A	Prophylaxis was carried out with a Magnetostrictive scaler working at a speed of 30 kHz, with a water pressure of 0.3 MPa during each treatment. A high speed air turbine	Prophylaxis was carried out with a Magnetostrictive scaler working at a speed of 30 kHz, with a water pressure of 0.3 MPa during each treatment. A high speed air turbine	Yes	No	N/A	Yes	Operator: chest Patient: chest

			handpiece, working at a speed of 400,000 rpm and with an air drive pressure of 0.25 MPa was used for preparing cavities on carious teeth.	handpiece, working at a speed of 400,000 rpm and with an air drive pressure of 0.25 MPa was used for preparing cavities on carious teeth.					
63	**Rautemaa 2006**	40 minutes	High speed rotating instrument (for the restorative treatment). No equipment details were provided regarding (endodontic/periodontic) treatment	Not stated	N/A	No	N/A	Yes	Operator: mask Dental Assisstant: mask
88	**Samaranayake 1989**	5-15 mins.	Not stated	Not stated	Yes	Not stated	Not stated	No	N/A
128	**Shahdad 2021**	20 minutes	air turbine (W&H Synea Turbine TA-98LED, Bürmoos, Austria)	360.000rpm	Yes	Yes	high-volume suction (HVS) and a saliva ejector (SE).	No	NA
94	**Tag El-Din 1997**	5-15 minutes	air-turbine-driven handpiece	Air driven	Yes	Yes	with and without rubber dam	Yes	Patient: chest
87	**Yamada 2011**	Not stated	High speed rotating instrument and ultrasonic scaler	Not stated	Yes	Yes	High volume evacuator systems (2 at different locations)	No	N/A

Unique Study ID	Study author and reference	If the duration of the procedure was state	If applicable, what was the main equipment used in performing the procedure?	If stated and considered relevant, what was the working	Is the equipment used in performing the dental procedu	Was any additional equipments used as a	If yes, Please specify in here	Person e.g. dental operator, dental nurse/assista	If Yes please specify who and which parts of body	Environment area measured within surgery or laboratory was it air, ? (choose	If Yes, please specify which areas of the environment	Was the measure microbiological? (Please choose	Please state the microbiological type (bacteria,	If the organism measured

		d, please enter it here	(e.g.rotary handpiece, ultrasonic scaler)	mechanism and/or the rotation /vibration speed of the main equipment used	re relevant to today's practice (Please choose from the dropdown list Yes, No, N/A)	standard measure e.g. rubber dam, suction) (Please choose from the dropdown list Yes, No, N/A)		nt, patient (choose from dropdown list; Yes, No, N/A)		from dropdown list; Yes, No, N/A)		from the dropdown list Yes/ other)	viruses, fungi, prions)	was stated specifically, please state here (e.g. aerobic bacteria, respiratory virus, Hep B, HIV, aspergilla etc)
99	**Ahmed 2021**	**3** minutes	Two-hole AA87handpiece (PANA MAX, NSK, Tochigi, Japan) - four-hole handpiece (KAVO, Biberach, Germany).	high-speed handpiece	Yes	Yes	High volume suction (DSEplus Type 5193, KAVO,Wart hausen, Germany)	Yes	operator chest, head, forearms, upper leg, and inside facemask. assistant: chest, head, forearms, upper leg, and inside facemask.	Yes	Multiple qualitative cotton cellulose filter paper (Whatman; Maidstone, England) was placed at a distance of 12-inches from each other up to 60-inches away from the dental manikin and at six different directions (at 2, 4, 6, 8, 10, and	Other	N/A	N/A

											12 o'clock positions) around the maninkin head .			
100	**Allison 2021(a)**	**High Speed** Air Turbine = 10mins 3-in-1 Spray = 30 seconds (replicate washing acid etchant)	Air turbine - Synea TA-98, W&H (UK) LTD and 3-in-1 Spray = Model not stated	high-speed handpiece. 3-in1 = air and water 140.6mL / min	yes	Yes	Suction for 3-in-1 measure at 6.3L of water per minute. Measured with and without suction for high speed	Yes - simulated body of Mannequin and Operator and Assistant	Above mannequin mouth. 4 papers on body of mannequin x2 40cm from hub and x2 80cm.<br><br>**Assistant** and Operator - upper mid forearm, upper chest, upper mid-thigh, Full-face visor and vertex of the head. Additionally for anterior crown prep with suction plus assistant, 3 filter papers were placed on the mask (beneath a full-face visor).	Yes	Platform spaced at 0.5, intervals along eight, 4m, rigid rods, laid out at 45 degree intervals and supported by a cetral hub, thus creating a 8m diameter circle around the mannequin. The centre of the circle was placed 25cm superior to the mouth. Aerosol & splatter measured by contaminated surface area distance from centre 0, 0.5, 1, 1.5, 2m)	Other	N/A	N/A
120	**Allison 2021(b)**	**10 minutes**	A speed-increasing handpiece (no water) High-speed	Highspeed: Approx: 400.000rpm<br><br>Micromotor: 60.000rpm; 120.000rpm; 200.000rpm	yes	yes	suction (flow rate 105l/min)	Yes - simulated body of Mannequin and Operator and Assistant	Above mannequin mouth. 4 papers on body of mannequin x2 40cm from hub and x2 80cm. Assistant and Operator - upper mid forearm, upper chest, upper mid-thigh, Full-face visor and vertex of the head. Additionally for anterior crown prep with suction plus assistant, 3 filter papers were placed on the mask (beneath a full-face visor).	only measured settle on papers	Platform spaced at 0.5, intervals along eight, 4m, rigid rods, laid out at 45 degree intervals and supported by a cetral hub, thus creating a 8m diameter circle around the mannequin. The centre of the circle was placed 25cm superior to the mouth. Aerosol & splatter measured by	Other	N/A	N/A

											contaminated surface area distance from centre 0, 0.5, 1. 1.5, 2m)			
127	**Allison 2021(c)**	**10** minutes	High-speed air-turbine (Synea TA-98, W&H (UK) Ltd.; St Albans, UK).	no	yes	yes	medium volume suction (dental suction with an 8.3 mm internal diameter suction tip at a flow rate of 133 L/min)	Yes	Active air sampling: **Operato**r: the left chest pocket	Air sampling and settled filter paper	Air sampling: Particle counting and multiple-chair setting: 0.5 m inferior to the mouth of the mannequin, and to the left of the mannequin at 2m. In single chair setting: 0.5 m to the right of the mannequin and 2m at the foot of the dental chair.<br><br>Active sampling: Multiple-chair setting: 0.5, 1.0, and 2.0 m to the left of the mannequin. Single surgery setting: at 0.15 m on the mannequin (chest), 0.5 m to the right of the mannequin, 1 m on the dental chair, and 2 m at the end of the dental chair.<br><br>Passive sampling: filter papers fixed on a platform and spaced at 0.5,	No	N/A	N/A

											intervals along eight, 4m, rigid rods, laid out at 45 degree intervals and supported by a cetral hub, thus creating a 8m diameter circle around the mannequin. The centre of the circle was placed 25cm superior to the mouth. Aerosol & splatter measured by contaminated surface area distance from centre 0, 0.5, 1. 1.5, 2m)			
93	**Belting 1964**	**1** minute	Air rotor	The dental air rotor (Borden Airotor, Ritter Co., Inc., Rochester, N. Y.) with no. 171 bur attached operating at an air flow pressure of 30 psi using 30 drops of oil and 18ml water	YES?	Yes	air rotor was used with and without water	No	N/A	Yes	Petri dishes placed in three positions: (1) Infront of the patient mouth at chin level 6 inches away (2) Bracket table in front of the patient, 2 ft. away from patient mouth, (3) On the instrument cabine to the right front of the patient 4 ft. away from patient mouth.	**Yes**	Bacterial	Mycobacterium tuberculosis

				per minute										
8	**Bentley 1994**	**Experiment 1) 2 minutes (spatter)** Experiment 2a) 30 minutes (aerosol with high speed Experiment 2b))30 minutes (aerosol with ultrasonic)	1) high speed handpiece 2 high speed handpiece	Not stated	**Yes**	Yes	1) High volume aspirator with high speed handpiece tooth preparation 2a) high volume aspirator with high speed handpiece 2b) saliva ejector with ultrasonic scaler	Yes	**Operator:** headcaps, masks and gowns. **Dental Assistant**: headcaps, masks and gowns. **Patient**: chest	Yes	1) Blood agar culture plates were placed along the six spokes of the headrest extension device at 12 and 24 inches from the subject's mouth 2) bracket table, 3) counter tops, 4) light	Both	Bacterial	alpha haemolytic streptococci
29	**Grenier 1995**	**High-speed** drilling= 8 minutes	High speed handpiece	not stated	**Yes**	Yes	rubber dam	**No**	N/A	Air sampling	Air sampler placed 122 cm away from the patient's mouth	**Yes**	Bacterial	anaerobic bacteria
122	**Grzech-Lesniak 2021**	**NA**	highspeed handpiece W&H Synea TA-98LC	200,000rpm 15,000rpm	Yes	Yes	1) saliva ejector (SE) EM15 (Monoart®	Yes	Operatore mouth: 2 cm away Assistant: Mouth : 2cm	No	NA	Other	N/A	N/A

			(W&H, Bürmoos, Austria) low-speed handpiece W&H Synea TA-98LC (W&H, Bürmoos, Austria)				Euronda, Vicenza, Italy) and (2) high-volume evacuator (HVE) EM19 EVO (Monoart® Euronda, Vicenza, Italy). Evacuators were placed at the level of the tooth around 2 cm from its buccal side.		away<br>Patient (simulated): mputh					
107	**Han 2021**	**Low** speed : 5 min Highspeed: 5s triple syringe: 15s	Air turbine handpiece ((TiMax, NSK, Kanuma, Japan) low-speed air turbine handpiece (W&H implantMED™, Bürmoos, Austria)<br><br>Triple syringe	300,000 rpm 1200 rpm	Yes	None	N/A	Yes	**Operator:** 20cm representing dentist position<br>**Assisstant**: 29 cm representing the Assistant position	Yes	3:35cm opposite the dentist<br>4: 29cm away from the left chest<br>5: 30 cm in the middle<br>6: 29cm away from the right chest<br>7: 60 cm in front of the patient mouth (left)<br>8 : 60cm in front of the patient mouth (right)<br>9: 80 cm away from the patient (left)<br>10 120cm away from the patient (right)	Other	NA	NA

36	**Hausler 1966**	**not** clear	high speed handpiece and air turbine	2.000 rpm	No	No	This is an old study, here is relevance bt the handpieces etc may not be the same	**No**	NA	Air sampling+ Settle plates	Three air samplers and plates were placed in a straight line at distance of 10, 20, and 30 inches from the tooth.	**Yes**	Bacteria l	s mar cenc ens
108	**Holliday 2021**	**10 minut es**	Highspeed Hand piece.	not stated	Yes	Dental suctio n at two flow rates: low volum e suctio n, 40 L/min of air; mediu m volum e suctio n, 159 L/min of air.		**No**	na	Yes	***Around the mannequin:** 1 m diameter rig was constructed with four rods arranged at 90◦<br><br>***Further away within the dental setting:** A linear 12m rig was constructed in the adjacent walkway which had collection platforms at 0.5 m intervals for the 2 m either side of the centre, and then subsequently at 1 m intervals out to 6 m either side of the centre.<br><br>*Benchtops (86 cm height);<br>*bracket table situated directly over the dental chair;<br>*On top of separating divides to the other half of the clinic where appropriate (123cm height).	Other	NA	NA

109	**Ionescu 2020**	**4** minutes	* Highspeed: Air turbine handpiece (Bora Led, Bien-Air Dental) equipped with a cylindrical diamond bur (835KR.314.016, Komet Italia Srl).<br><br>**Slow-speed: contra-angle handpiece (CA 1:1, Bien-Air) with a round tungsten carbide bur (H1SM.204.020, Komet) inside the already prepared cavity.	320,000 rpm. 50.000 rpm	**Yes**	N/A	N/A	Yes	**Patient:** 14 sites on the dental chair<br>**Assisstant**: 1 on the assistant pad	Yes	Dental chair unit: instrument tray, cuspidor cup, water glass tray, overhead dental unit light, foot pedal.<br>Floor : 60,120,180, 240,360<br>Wall in front (distance not clear, >1).<br>The lateral column (distance not clear)<br>The back wall (distance not clear)<br>Ceiling (distance not clear, >1<br>the cabinet (distance not clear)	Yes	Bacteria	Streptoccus mutans
91	**Larato 1966**	**1.5-5** mins.	air turbine drill	N/A	**YES?**	Yes	Unit aspirating system	N/A	N/A	Air sampling	Air Sampler placed on the bracket table 15 in. (anterior) to and slightly below the patient's mouth	**Yes**	Bacterial	Hemolytic Staphylococcus albus, Nonhemolytic

														Staphylococcus albus, Alpha streptococcus (three most common/were exist in all of the sample)
113	**Llandro 2021**	**10** minutes	* speed-increasing dental handpiece driven by a dental air motor at full speed (with no water coolant)		Yes	yes	Large bore dental suction	Yes	**Operator:** forearms, chest, upper leg and head, their masks and full-face visor. **assisstant:** forearms, chest, upper leg and head, their masks and full-face visor.	Yes	Eight-metre diameter around mannequin head . The filter papers were spaced at intervals of 0.5 m on eight four metre rods, arranged at 45-degree intervals around a dental training mannequin	Other	na	na
50	**Manarte - Monteiro 2013**	**1-4** hours	Manual (endodontic) + high-speed handpieces	rotary action, with	**Yes**	Yes	Rubber dam	NA	NA	Yes	Blood agar plates were placed at 1) 0.5 meter	**Yes**	Bacterial	Gram-positive

			(turbine) (for (endodontic & restorative)	water-cooling;							2) 2 meter from the patient head position			cocci
90	Miller 1971	20 seconds	Air turbine Handspeed+ 557 bur (multible procedures). Rubber cup and pumice (periodontics) .	N/A	YES?	Yes	High velocity suction	No	N/A	Yes	Five wooded battens were installed on a plane 0.92 meter (3 feet) above the floor in a pattern radiating from a point 0.304 meter (1 foot) below the patient's mouth to the sides and end of the room. These battens were mounted to rotate on their long axes and were fitted with suction cups at 0.304 meter (1 foot) intervals along their lengths.	Yes	Bacterial	N/A
115	Nulty 2020	1 Minutes	Electric Micromotor: " Micromotor high-speed handpiece with water"Mirco motor Air turbine: HIGH SPEED WITH WATER Low speed with water Three-in-one		Yes	yes	saliva ejector	no	N/A	yes	air sampler placed 420 mm to the right of the phantom head	Other	na	na
60	Purohit 2009	N/A	Prophylaxis was carried out with a Magnetostrictive scaler	Prophylaxis was carried out with a	Yes	No	N/A	Yes	**Operator**: chest Patient: chest	Yes	Mimicking Bentley et al. model (ID: 8) : headrest extension device at a distance of 12 inches and 24	Yes	Bacterial	N/A

			working at a speed of 30 kHz, with a water pressure of 0.3 MPa during each treatment. A high speed air turbine handpiece, working at a speed of 400,000 rpm and with an air drive pressure of 0.25 MPa was used for preparing cavities on carious teeth.	Magnetostrictive scaler working at a speed of 30 kHz, with a water pressure of 0.3 MPa during each treatment. A high speed air turbine handpiece, working at a speed of 400,000 rpm and with an air drive pressure of 0.25 MPa was used for preparing cavities on carious teeth.							inches away from the operating area			

63	**Rautema a 2006**	**40 minutes**	High speed rotating instrument (for the restorative treatment). No equipment details were provided regarding (endodontic/periodontic) treatment	Not stated	**N/**A	No	N/A	**Yes**	**Operator:** mask<br>Dental Assisstant: mask	Yes	Plates were placed in six different sectors 0.5- 2 meter from the patient:<br><br>1) 2 plates in front of the patient at a distance of 2 meter.<br>2) 1 plate behind the patient at 0.5 meter.<br>3) 1 plate behind assissnt side (patient left hand side) at 1.5 meter.<br>4) 2 plates behind the operator side (patient right hand side) at 1.5 meter and the surgery compture location (no distance reported).	**Yes**	Bacterial	airborne
88	**Samaranayake 1989**	**5-15** mins.	Not stated	Not stated	**Yes**	Not stated	Not stated	**No**	N/A	Yes	1, 2 and 3 meters from the headrest of the dental chair	**Yes**	Bacterial	Airborne
128	**Shahdad 2021**	**20 minutes**	air turbine (W&H Synea Turbine TA-98LED, Bürmoos, Austria)	360.000rpm	Yes	Yes	high-volume suction (HVS) and a saliva ejector (SE).	No	NA	Air Sampling	The sampling inlets placed adjacent to the manikin's mouth in the 7 o'clock position (8 cm from tooth 21	No	N/A	N/A
94	**Tag El-Din 1997**	**5-15** minutes	air-turbine-driven handpiece	Air driven	**Yes**	Yes	with and without rubber dam	**Yes**	**Patient:** chest	Yes	1) 3 plates on the left and right sides and behind the patient (All placed equidistantly from the child's head).<br><br>2) 1 metre; and	**Yes**	Bacterial	Not stated

											3) 2 metres from the head-rest of the dental chair (to further details on distance were reported).			
87	**Yamada 2011**	Not stated	High speed rotating instrument and ultrasonic scaler	Not stated	**Yes**	Yes	High volume evacuator systems (2 at different locations)	**No**	N/A	Air sampling	Blood contaminated aerosol realeased in the atmospheric air 1) 50 cm away from patient mouth. 2) 100 cm away from patient mouth.	Other	N/A	N/A

Unique Study ID	Study author and reference	If the duration of the procedure was stated, please enter it here	If applicable, what was **the main equipment used in performing the procedure? (e.g.rotary handpiece, ultrasonic scaler)**	If stated and considered relevant, what was the working mechanism and/or the rotation/vibration **speed of the main equipment used**	Is the equipment used in performing the dental procedure relevant to today's practice (Please choose from the dropdown list Yes, No, N/A)	Was any additional equipments used as a **standard measure e.g. rubber dam, suction)** (Please choose from the dropdown list Yes, No, N/A)	If yes, Please specify in here	Person e.g. dental operator, dental nurse/ assistant, patient (choose **from dropdown list; Yes, No, N/A)**
99	**Ahmed 2021**	3 minutes	**Two-hole** AA87handpiece (PANA MAX, NSK, Tochigi, Japan) - four-hole handpiece (KAVO, Biberach, Germany).	high-speed handpiece	Yes	Yes	High volume suction (DSEplus Type 5193, KAVO,Warthausen, Germany)	Yes
100	**Allison 2021(a)**	High Speed Air Turbine = 10mins 3-in-1 Spray = 30 seconds (replicate washing acid etchant)	Air turbine - Synea TA-98, W&H (UK) LTD and 3-in-1 Spray = Model not stated	high- speed handpiece. 3-in1 = air and water 140.6mL / min	yes	Yes	Suction for 3-in-1 measure at 6.3L of water per minute. Measured with and without suction for high speed	Yes - simulated body of Mannequin and Operator and Assistant

120	**Allison 2021(b)**	**10 minutes**	**A speed-increasing handpiece *(no water)* *High-speed***	Highspeed: Approx: 400.000rpm<br><br>Micromotor: 60.000rpm; 120.000rpm; 200.000rpm	yes	yes	suction (flow rate 105l/min)	Yes - simulated body of Mannequin and Operator and Assistant
127	**Allison 2021(c)**	**10** minutes	**High-speed** air-turbine (Synea TA-98, W&H (UK) Ltd.; St Albans, UK).	no	yes	yes	medium volume suction (dental suction with an 8.3 mm internal diameter suction tip at a flow rate of 133 L/min)	Yes
93	**Belting 1964**	**1** minute	**Air** rotor	The dental air rotor (Borden Airotor, Ritter Co., Inc., Rochester, N. Y.) with no. 171 bur attached operating at an air flow pressure of 30 psi using 30 drops of oil and 18ml water per minute	YES?	Yes	air rotor was used with and without water	No
8	**Bentley 1994**	**Experiment** 1) 2 minutes (spatter) Experiment 2a) 30 minutes (aerosol with high speed Experiment 2b))30 minutes (aerosol with ultrasonic)	**1)** high speed handpiece 2 high speed handpiece	Not stated	Yes	Yes	1) High volume aspirator with high speed handpiece tooth preparation 2a) high volume aspirator with high speed handpiece 2b) saliva ejector with ultrasonic scaler	Yes
29	**Grenier 1995**	**High-speed** drilling= 8 minutes	**High** speed handpiece	not stated	Yes	Yes	rubber dam	No
122	**Grzech-Lesniak 2021**	**NA**	**highspeed handpiece W**&H Synea TA-98LC (W&H, Bürmoos, Austria)	200,000rpm 15,000rpm	Yes	Yes	1) saliva ejector (SE) EM15 (Monoart® Euronda, Vicenza,	Yes

			low-speed handpiece W&H Synea TA-98LC (W&H, Bürmoos, Austria)				Italy) and (2) high-volume evacuator (HVE) EM19 EVO (Monoart® Euronda, Vicenza, Italy). Evacuators were placed at the level of the tooth around 2 cm from its buccal side.	
107	**Han 2021**	**Low** speed: 5 min<br>Highspeed: 5s<br>triple syringe: 15s	**Air turbine handpiece** ((TiMax, NSK, Kanuma, Japan)<br>low-speed air turbine handpiece (W&H implantMED™, Bürmoos, Austria)<br><br>Triple syringe	300,000 rpm<br>1200 rpm	Yes	None	N/A	Yes
36	**Hausler 1966**	**not** clear	**high speed** handpiece and air turbine	2.000 rpm	No	No	This is an old study, here is relevance bt the handpieces etc may not be the same	No
108	**Holliday 2021**	**10** minutes	**Highspeed** Hand piece.	not stated	Yes	Dental suction at two flow rates: low volume suction, 40 L/min of air; medium volume suction, 159 L/min of air.		No
109	**Ionescu 2020**	**4** minutes	*** Highspeed: *Air turbine handpiece (Bora* Led, Bien-Air** Dental) equipped with a cylindrical diamond bur (835KR.314.016, Komet Italia Srl).<br><br>**Slow-speed: contra-	320,000 rpm.<br>50.000 rpm	Yes	N/A	N/A	Yes

			angle handpiece (CA 1:1, Bien-Air) with a round tungsten carbide bur (H1SM.204.020, Komet) inside the already prepared cavity.					
91	**Larato 1966**	**1.5-5** mins.	**air** turbine drill	N/A	YES?	Yes	Unit aspirating system	N/A
113	**Llandro 2021**	**10 minutes**	**** speed-increasing dental handpiece driven by a dental air motor at full speed (with no water coolant)***		Yes	yes	Large bore dental suction	Yes
50	**Manarte-Monteiro 2013**	**1-4** hours	**Manual** (endodontic) + high-speed handpieces (turbine) (for (endodontic & restorative)	rotary action, with water-cooling;	Yes	Yes	Rubber dam	NA
90	**Miller 1971**	**20 seconds**	**Air** turbine Handspeed+ 557 bur (multible procedures). Rubber cup and pumice (periodontics).	N/A	YES?	Yes	High velocity suction	No
115	**Nulty 2020**	**1** Minutes	**Electric Micromotor: "** ***Micromotor high-speed handpiece with water"*****Mircomotor** **Air turbine:** HIGH SPEED WITH WATER Low speed with water Three-in-one		Yes	yes	saliva ejector	no
60	**Purohit 2009**	**N/**A	**Prophylaxis** was carried out with a Magnetostrictive scaler working at a speed of 30 kHz, with a water pressure of 0.3 MPa	Prophylaxis was carried out with a Magnetostrictive scaler working at a speed of 30 kHz, with a water pressure of 0.3 MPa during each treatment. A high speed air turbine handpiece,	Yes	No	N/A	Yes

			during each treatment. A high speed air turbine handpiece, working at a speed of 400,000 rpm and with an air drive pressure of 0.25 MPa was used for preparing cavities on carious teeth.	working at a speed of 400,000 rpm and with an air drive pressure of 0.25 MPa was used for preparing cavities on carious teeth.				
63	**Rautemaa 2006**	**40 minutes**	**High** speed rotating instrument (for the restorative treatment). No equipment details were provided regarding (endodontic/periodontic) treatment	Not stated	N/A	No	N/A	Yes
88	**Samaranayake 1989**	**5-15** mins.	**Not** stated	Not stated	Yes	Not stated	Not stated	No
128	**Shahdad 2021**	**20** minutes	**air** turbine (W&H Synea Turbine TA-98LED, Bürmoos, Austria)	360.000rpm	Yes	Yes	high-volume suction (HVS) and a saliva ejector (SE).	No
94	**Tag El-Din 1997**	**5-15 minutes**	**air-turbine-driven** handpiece	Air driven	Yes	Yes	with and without rubber dam	Yes
87	**Yamada 2011**	**Not** stated	**High** speed rotating instrument and ultrasonic scaler	Not stated	Yes	Yes	High volume evacuator systems (2 at different locations)	No

**Unique Study ID**	**Study author and reference**	**If the duration of the procedure was stated, please enter it here**	**If applicable, what was the main equipment used in performing the procedure? (e.g.rotary handpiece, ultrasonic scaler)**	**If stated and considered relevant, what was the working mechanism and/or the rotation/vibration speed of the main equipment used**	**Is the equipment used in performing the dental procedure relevant to today's practice (Please choose** from the dropdown list Yes, No, N/A)	**Was any additional equipments used as a standard measure e.g. rubber dam, suction)** (Please choose from the dropdown list Yes, No, N/A)

**99**	**Ahmed 2021**	3 minutes	Two-hole AA87handpiece (PANA MAX, NSK, Tochigi, Japan)<br>- four-hole handpiece (KAVO, Biberach, Germany).	high-speed handpiece	Yes	Yes
**100**	**Allison 2021(a)**	High Speed Air Turbine = 10mins 3-in-1 Spray = 30 seconds (replicate washing acid etchant)	Air turbine - Synea TA-98, W&H (UK) LTD and 3-in-1 Spray = Model not stated	high- speed handpiece. 3-in1 = air and water 140.6mL / min	yes	Yes
**120**	**Allison 2021(b)**	10 minutes	A speed-increasing handpiece (no water)<br>High-speed	Highspeed: Approx: 400.000rpm<br><br>Micromotor:<br>60.000rpm;<br>120.000rpm;<br>200.000rpm	yes	yes
**127**	**Allison 2021(c)**	10 minutes	High-speed air-turbine (Synea TA-98, W&H (UK) Ltd.; St Albans, UK).	no	yes	yes
**93**	**Belting 1964**	1 minute	Air rotor	The dental air rotor (Borden Airotor, Ritter Co., Inc., Rochester, N. Y.) with no. 171 bur attached operating at an air flow pressure of 30 psi using 30 drops of oil and 18ml water per minute	YES?	Yes

8	**Bentley 1994**	Experiment 1) 2 minutes (spatter) Experiment 2a) 30 minutes (aerosol with high speed Experiment 2b))30 minutes (aerosol with ultrasonic)	1) high speed handpiece 2 high speed handpiece	Not stated	Yes	Yes
29	**Grenier 1995**	High-speed drilling= 8 minutes	High speed handpiece	not stated	Yes	Yes
122	**Grzech-Lesniak 2021**	NA	highspeed handpiece W&H Synea TA-98LC (W&H, Bürmoos, Austria)<br>low-speed handpiece W&H Synea TA-98LC (W&H, Bürmoos, Austria)	200,000rpm<br>15,000rpm	Yes	Yes
107	**Han 2021**	Low speed: 5 min<br>Highspeed: 5s<br>triple syringe: 15s	Air turbine handpiece ((TiMax, NSK, Kanuma, Japan)<br>low-speed air turbine handpiece (W&H implantMED™, Bürmoos, Austria)<br><br>Triple syringe	300,000 rpm<br>1200 rpm	Yes	None
36	**Hausler 1966**	not clear	high speed handpiece and air turbine	2.000 rpm	No	No

108	**Holliday 2021**	10 minutes	Highspeed Hand piece.	not stated	Yes	Dental suction at two flow rates:<br>low volume suction, 40 L/min of air;<br>medium volume suction, 159 L/min of air.
109	**Ionescu 2020**	4 minutes	* Highspeed: Air turbine handpiece (Bora Led, Bien-Air Dental)<br>equipped with a cylindrical diamond bur (835KR.314.016, Komet Italia Srl).<br><br>**Slow-speed: contra-angle handpiece (CA 1:1, Bien-Air) with a round tungsten carbide bur (H1SM.204.020, Komet) inside the already prepared cavity.	320,000 rpm.<br>50.000 rpm	Yes	N/A
91	**Larato 1966**	1.5-5 mins.	air turbine drill	N/A	YES?	Yes
113	**Llandro 2021**	10 minutes	* speed-increasing dental handpiece driven by a dental air motor at full speed (with no water coolant)		Yes	yes
50	**Manarte-Monteiro 2013**	1-4 hours	Manual (endodontic) + high-speed handpieces (turbine) (for (endodontic & restorative)	rotary action, with water-cooling;	Yes	Yes
90	**Miller 1971**	20 seconds	Air turbine Handspeed+ 557 bur (multible procedures). Rubber cup and pumice (periodontics).	N/A	YES?	Yes

115	**Nulty 2020**	1 Minutes	Electric Micromotor: " Micromotor high-speed handpiece with water"Mircomotor<br>Air turbine: HIGH SPEED WITH WATER<br>Low speed with water<br>Three-in-one		Yes	yes
60	**Purohit 2009**	N/A	Prophylaxis was carried out with a Magnetostrictive scaler working at a speed of 30 kHz, with a water pressure of 0.3 MPa during each treatment. A high speed air turbine handpiece, working at a speed of 400,000 rpm and with an air drive pressure of 0.25 MPa was used for preparing cavities on carious teeth.	Prophylaxis was carried out with a Magnetostrictive scaler working at a speed of 30 kHz, with a water pressure of 0.3 MPa during each treatment. A high speed air turbine handpiece, working at a speed of 400,000 rpm and with an air drive pressure of 0.25 MPa was used for preparing cavities on carious teeth.	Yes	No
63	**Rautemaa 2006**	40 minutes	High speed rotating instrument (for the restorative treatment). No equipment details were provided regarding (endodontic/periodontic) treatment	Not stated	N/A	No
88	**Samaranayake 1989**	5-15 mins.	Not stated	Not stated	Yes	Not stated
128	**Shahdad 2021**	20 minutes	air turbine (W&H Synea Turbine TA-98LED, Bürmoos, Austria)	360.000rpm	Yes	Yes
94	**Tag El-Din 1997**	5-15 minutes	air-turbine-driven handpiece	Air driven	Yes	Yes
87	**Yamada 2011**	Not stated	High speed rotating instrument and ultrasonic scaler	Not stated	Yes	Yes

Unique Study ID	Study author and reference	If the duration of the procedure was stated, please enter it here	If applicable, what was the main equipment used in performing the procedure? (e.g.rotary handpiece, ultrasonic scaler)	If stated and considered relevant, what was the working mechanism and/or the rotation/vibration speed of the main equipment used	Is the equipment used in performing the dental procedure relevant to today's practice (Please choose from the dropdown list Yes, No, N/A)
99	**Ahmed 2021**	3 minutes	Two-hole AA87handpiece (PANA MAX, NSK, Tochigi,<br>Japan)<br>- four-hole handpiece (KAVO, Biberach, Germany).	high-speed handpiece	Yes
100	**Allison 2021(a)**	High Speed Air Turbine = 10mins 3-in-1 Spray = 30 seconds (replicate washing acid etchant)	Air turbine - Synea TA-98, W&H (UK) LTD and 3-in-1 Spray = Model not stated	high- speed handpiece. 3-in1 = air and water 140.6mL / min	yes
120	**Allison 2021(b)**	10 minutes	A speed-increasing handpiece (no water)<br>High-speed	Highspeed: Approx: 400.000rpm<br><br>Micromotor: 60.000rpm;<br>120.000rpm;<br>200.000rpm	yes
127	**Allison 2021(c)**	10 minutes	High-speed air-turbine (Synea **TA-98,** W&H (UK) Ltd.; St Albans, UK).	no	yes
93	**Belting 1964**	1 minute	Air rotor	The dental air rotor (Borden Airotor, Ritter Co., Inc., Rochester, N. Y.) with no. 171 bur attached operating at an air flow pressure of 30 psi	YES?

				using 30 drops of oil and 18ml water per minute	
8	Bentley 1994	Experiment 1) 2 minutes (spatter) Experiment 2a) 30 minutes (aerosol with high speed Experiment 2b))30 minutes (aerosol with ultrasonic)	**1)** high speed handpiece 2 high **speed handpiece**	Not stated	Yes
29	Grenier 1995	High-speed drilling= 8 minutes	High speed handpiece	not stated	Yes
122	Grzech-Lesniak 2021	NA	highspeed handpiece W&H Synea TA-98LC (W&H, Bürmoos, Austria)<br>low-speed handpiece W&H Synea TA-98LC (W&H, Bürmoos, Austria)	200,000rpm<br>15,000rpm	Yes
107	Han 2021	Low speed: 5 min<br>Highspeed: 5s<br>triple syringe: 15s	**Air** turbine handpiece ((TiMax, **NSK**, Kanuma, Japan)<br>low-speed air turbine handpiece (W&H implantMED™, Bürmoos, Austria)<br><br>Triple syringe	300,000 rpm<br>1200 rpm	Yes
36	Hausler 1966	not clear	high speed handpiece and air turbine	2.000 rpm	No
108	Holliday 2021	10 minutes	Highspeed Hand piece.	not stated	**Yes**
109	Ionescu 2020	4 minutes	* **Highspeed**: Air turbine handpiece (Bora Led**,** Bien-Air Dental)<br>equipped with a cylindrical diamond bur (835KR.314.016, Komet Italia Srl).<br><br>**Slow-speed: contra-angle handpiece (CA 1:1, Bien-Air) with a round tungsten	320,000 rpm.<br>50.000 rpm	Yes

			carbide bur (H1SM.204.020, Komet) inside the already prepared cavity.		
91	Larato 1966	1.5-5 mins.	air turbine drill	N/A	YES?
113	Llandro 2021	10 minutes	* speed-increasing dental handpiece driven by a dental air motor at full speed (with **no water** coolant)		Yes
50	Manarte-Monteiro 2013	1-4 hours	Manual (endodontic) + high-speed handpieces (turbine) (for (endodontic & restorative)	rotary action, with water-cooling;	Yes
90	Miller 1971	20 seconds	Air turbine Handspeed+ 557 bur (multible procedures). Rubber cup and pumice (periodontics).	N/A	YES?
115	Nulty 2020	1 Minutes	Electric Micromotor: " Micromotor high-speed handpiece with water"Mircomotor<br>Air turbine: HIGH SPEED WITH WATER<br>Low speed with water<br>Three-in-one		Yes
60	Purohit 2009	N/A	**Prophylaxis** was carried out with a Magnetostrictive scaler working at a speed of 30 kHz, with a water pressure of 0.3 MPa during each treatment. A high speed air turbine handpiece, working at a speed of 400,000 rpm and with an air drive pressure of 0.25 MPa was used for preparing cavities on carious teeth.	Prophylaxis was carried out with a Magnetostrictive scaler working at a speed of 30 kHz, with a water pressure of 0.3 MPa during each treatment. A high speed air turbine handpiece, working at a speed of 400,000 rpm and with an air drive pressure of 0.25 MPa was	Yes

				used for preparing cavities on carious teeth.	
63	Rautemaa 2006	40 minutes	**High** speed rotating instrument (for the restorative treatment). No equipment details were provided regarding (endodontic/periodontic) treatment	Not stated	N/A
88	Samaranayake 1989	5-15 mins.	Not stated	Not stated	Yes
128	Shahdad 2021	20 minutes	air turbine (W&H Synea Turbine TA-98LED, Bürmoos, Austria)	360.000rpm	Yes
94	Tag El-Din 1997	5-15 minutes	**air-turbine-driven** handpiece	Air driven	Yes
87	Yamada 2011	Not stated	High speed rotating instrument and ultrasonic scaler	Not stated	Yes

Unique Study ID	Study author and reference	If the duration of the procedure was stated, please enter it here	If applicable, what was the main equipment used in performing the procedure? (e.g.rotary handpiece, ultrasonic scaler)	If stated and considered relevant, what was the working mechanism and/or the rotation/vibration speed of the main equipment used	Is the equipment used in performing the dental procedure relevant to today's practice (Please choose from the	Was any additional equipments used as a standard measure e.g. rubber dam, suction) (Please choose from the	If yes, Please specify in here	Person e.g. dental operator, dental nurse/ assistant, patient (choose from dropdown list; Yes, No, N/A)	If Y es please specify who and which parts of body

					dropdown list Yes, No, N/A)	dropdown list Yes, No, N/A)			
99	**Ahmed 2021**	3 minutes	Two-hole AA87handpiece (PANA MAX, NSK, Tochigi, Japan) - four-hole handpiece (KAVO, Biberach, Germany).	high-speed handpiece	Yes	Yes	High volume suction (DSEplus Type 5193, KAVO,Warthausen, Germany)	Yes	operator chest, head, forearms, upper leg, and inside facemask. assistant: chest, head, forearms, upper leg, and inside facemask.
100	**Allison 2021(a)**	High Speed Air Turbine = 10mins 3-in-1 Spray = 30 seconds (replicate washing acid etchant)	Air turbine - Synea TA-98, W&H (UK) LTD and 3-in-1 Spray = Model not stated	high- speed handpiece. 3-in1 = air and water 140.6mL / min	yes	Yes	Suction for 3-in-1 measure at 6.3L of water per minute. Measured with and without suction for high speed	Yes - simulated body of Mannequin and Operator and Assistant	Above mannequin mouth. 4 papers on body of mannequin x2 40cm from hub and x2 80cm. **Assistant** and Operator - upper mid

									forearm, upper chest, upper mid-thigh, Full-face visor and vertex of the head. Additionally for anterior crown prep with suction plus assistant, 3 filter papers were placed on the mask (beneath a full-face visor).
120	**Allison 2021(b)**	10 minutes	A speed-increasing handpiece (no water) High-speed	Highspeed: Approx: 400.000rpm<br><br>Micromotor: 60.000rpm; 120.000rpm; 200.000rpm	yes	yes	suction (flow rate 105l/min)	Yes - simulated body of Mannequin and Operator and Assistant	Above mannequin mouth. 4 papers on body of mannequin x2 40cm from hub and x2 80cm. Assistant and Operator - upper mid

									forearm, upper chest, upper mid-thigh, Full-face visor and vertex of the head. Additionally for anterior crown prep with suction plus assistant, 3 filter papers were placed on the mask (beneath a full-face visor).
127	**Allison 2021(c)**	10 minutes	High-speed air-turbine (Synea TA-98, W&H (UK) Ltd.; St Albans, UK).	no	yes	yes	medium volume suction (dental suction with an 8.3 mm internal diameter suction tip at a flow rate of 133 L/min)	Yes	Active air sampling: **Operato**r: the left chest pocket
93	**Belting 1964**	1 minute	Air rotor	The dental air rotor (Borden Airotor, Ritter Co., Inc., Rochester, N. Y.) with no. 171 bur	YES?	Yes	air rotor was used with and without water	No	N/A

				attached operating at an air flow pressure of 30 psi using 30 drops of oil and 18ml water per minute					
8	**Bentley 1994**	**Experiment** 1) 2 minutes (spatter) Experiment 2a) 30 minutes (aerosol with high speed Experiment 2b))30 minutes (aerosol with ultrasonic)	1) high speed handpiece 2 high speed handpiece	Not stated	Yes	Yes	1) High volume aspirator with high speed handpiece tooth preparation 2a) high volume aspirator with high speed handpiece 2b) saliva ejector with ultrasonic scaler	Yes	Operator: headcaps, masks and gowns. **Dental Assistant**: headcaps, masks and gowns. **Patient**: chest
29	**Grenier 1995**	**High-speed** drilling= 8 minutes	High speed handpiece	not stated	Yes	Yes	rubber dam	No	N/A

122	**Grzech-Lesniak 2021**	NA	highspeed handpiece W&H Synea TA-98LC (W&H, Bürmoos, Austria) low-speed handpiece W&H Synea TA-98LC (W&H, Bürmoos, Austria)	200,000rpm 15,000rpm	Yes	Yes	1) saliva ejector (SE) EM15 (Monoart® Euronda, Vicenza, Italy) and (2) high-volume evacuator (HVE) EM19 EVO (Monoart® Euronda, Vicenza, Italy). Evacuators were placed at the level of the tooth around 2 cm from its buccal side.	Yes	Operatore mouth: 2 cm away Assistant: Mouth : 2cm away Patient (simulated): mputh
107	**Han 2021**	Low speed: 5 min Highspeed: 5s triple syringe: 15s	Air turbine handpiece ((TiMax, NSK, Kanuma, Japan) low-speed air turbine handpiece (W&H implantMED™, Bürmoos, Austria)<br><br>Triple syringe	300,000 rpm 1200 rpm	Yes	None	N/A	Yes	Operator: 20cm representing dentist position **Assisstant**: 29 cm representing the Assistant position
36	**Hausler 1966**	not clear	high speed handpiece and air turbine	2.000 rpm	No	No	This is an old study, here is relevance bt the handpieces etc may not be the same	No	NA
108	**Holliday 2021**	10 minutes	Highspeed Hand piece.	not stated	Yes	Dental suction at two		No	na

						flow rates: low volume suction, 40 L/min of air; medium volume suction, 159 L/min of air.			
109	**Ionescu 2020**	4 minutes	* Highspeed: Air turbine handpiece (Bora Led, Bien-Air Dental) equipped with a cylindrical diamond bur (835KR.314.016, Komet Italia Srl).<br><br>**Slow-speed: contra-angle handpiece (CA 1:1, Bien-Air) with a round tungsten carbide bur (H1SM.204.020, Komet) inside the already prepared cavity.	320,000 rpm.<br>50.000 rpm	Yes	N/A	N/A	Yes	Patient: 14 sites on the dental chair **Assisstant**: 1 on the assistant pad

91	Larato 1966	1.5-5 mins.	air turbine drill	N/A	YES?	Yes	Unit aspirating system	N/A	N/A
113	Llandro 2021	10 minutes	* speed-increasing dental handpiece driven by a dental air motor at full speed (with no water coolant)		Yes	yes	Large bore dental suction	Yes	Operator: forearms, chest, upper leg and head, their masks and full-face visor. **assisstant:** forearms, chest, upper leg and head, their masks and full-face visor.
50	Manarte-Monteiro 2013	1-4 hours	Manual (endodontic) + high-speed handpieces (turbine) (for (endodontic & restorative)	rotary action, with water-cooling;	Yes	Yes	Rubber dam	NA	NA
90	Miller 1971	20 seconds	Air turbine Handspeed+ 557 bur (multible procedures). Rubber cup and pumice (periodontics).	N/A	YES?	Yes	High velocity suction	No	N/A

115	**Nulty 2020**	1 Minutes	Electric Micromotor: " Micromotor high-speed handpiece with water"Mircomotor<br>Air turbine: HIGH SPEED WITH WATER<br>Low speed with water<br>Three-in-one		Yes	yes	saliva ejector	no	N/A
60	**Purohit 2009**	N/A	Prophylaxis was carried out with a Magnetostrictive scaler working at a speed of 30 kHz, with a water pressure of 0.3 MPa during each treatment. A high speed air turbine handpiece, working at a speed of 400,000 rpm and with an air drive pressure of 0.25 MPa was used for preparing cavities on carious teeth.	Prophylaxis was carried out with a Magnetostrictive scaler working at a speed of 30 kHz, with a water pressure of 0.3 MPa during each treatment. A high speed air turbine handpiece, working at a speed of 400,000 rpm and with an air drive pressure of 0.25 MPa was used for preparing	Yes	No	N/A	Yes	Operator: chest Patient: chest

				cavities on carious teeth.					
63	Rautemaa 2006	40 minutes	High speed rotating instrument (for the restorative treatment). No equipment details were provided regarding (endodontic/periodontic) treatment	Not stated	N/A	No	N/A	Yes	Operator: mask Dental Assisstant: mask
88	Samaranayake 1989	5-15 mins.	Not stated	Not stated	Yes	Not stated	Not stated	No	N/A
128	Shahdad 2021	20 minutes	air turbine (W&H Synea Turbine TA-98LED, Bürmoos, Austria)	360.000rpm	Yes	Yes	high-volume suction (HVS) and a saliva ejector (SE).	No	NA
94	Tag El-Din 1997	5-15 minutes	air-turbine-driven handpiece	Air driven	Yes	Yes	with and without rubber dam	Yes	Patient: chest
87	Yamada 2011	Not stated	High speed rotating instrument and ultrasonic scaler	Not stated	Yes	Yes	High volume evacuator systems (2 at different locations)	No	N/A

Unique Study ID	Study author and reference	If the duration of the procedure was stated, please enter it here	If applicable, what was the main equipment used in performing the procedure? (e.g.rotary handpiece, ultrasonic scaler)	If stated and considered relevant, what was the working mechanism and/or the rotation/vibration speed of the	Is the equipment used in performing the dental procedure	Was any additional equipments used as a standard measure e.g. rubber dam, suction)	If yes, Please specify in here	Person e.g. dental operator, dental nurse/ assistant, patient (choose from dropdown list; Yes, No, N/A)

				main equipment used	relevant to today's practice (Please choose from the dropdown list Yes, No, N/A)	(Please choose from the dropdown list Yes, No, N/A)		
**99**	**Ahmed 2021**	3 minutes	Two-hole AA87handpiece (PANA MAX, NSK, Tochigi, Japan)<br>- four-hole handpiece (KAVO, Biberach, Germany).	high-speed handpiece	Yes	Yes	High volume suction (DSEplus Type 5193, KAVO,Warthausen, Germany)	Yes
100	**Allison 2021(a)**	High Speed Air Turbine = 10mins 3-in-1 Spray = 30 seconds (replicate washing acid etchant)	Air turbine - Synea TA-98, W&H (UK) LTD and 3-in-1 Spray = Model not stated	high- speed handpiece. 3-in1 = air and water 140.6mL / min	yes	Yes	Suction for 3-in-1 measure at 6.3L of water per minute. Measured with and without suction for high speed	Yes - simulated body of Mannequin and Operator and Assistant

120	**Allison 2021(b)**	10 minutes	A speed-increasing handpiece (no water)<br>High-speed	Highspeed: Approx: 400.000rpm<br><br>Micromotor: 60.000rpm; 120.000rpm; 200.000rpm	yes	yes	suction (flow rate 105l/min)	Yes - simulated body of Mannequin and Operator and Assistant
127	**Allison 2021(c)**	10 minutes	High-speed air-turbine (Synea TA-98, W&H (UK) Ltd.; St Albans, UK).	**no**	yes	yes	medium volume suction (dental suction with an 8.3 mm internal diameter suction tip at a flow rate of 133 L/min)	Yes
93	**Belting 1964**	1 minute	Air rotor	The dental air rotor (Borden Airotor, Ritter Co., Inc., Rochester, N. Y.) with no. 171 bur attached operating at an air flow pressure of 30 psi using 30 drops of oil and 18ml water per minute	YES?	Yes	air rotor was used with and without water	No
8	**Bentley 1994**	Experiment 1) 2 minutes (spatter) Experiment 2a) 30 minutes (aerosol with	1) high speed handpiece 2 high speed handpiece	Not stated	Yes	Yes	1) High volume aspirator with high speed handpiece tooth preparation 2a)	Yes

		high speed Experiment 2b))30 minutes (aerosol with ultrasonic)					high volume aspirator with high speed handpiece 2b) saliva ejector with ultrasonic scaler	
29	**Grenier 1995**	High-speed drilling= 8 minutes	High speed handpiece	not stated	Yes	Yes	rubber dam	No
122	**Grzech-Lesniak 2021**	NA	highspeed handpiece W&H Synea TA-98LC (W&H, Bürmoos, Austria)<br>low-speed handpiece W&H Synea TA-98LC (W&H, Bürmoos, Austria)	200,000rpm<br>15,000rpm	Yes	Yes	1) saliva ejector (SE) EM15 (Monoart® Euronda, Vicenza, Italy) and (2) high-volume evacuator (HVE) EM19 EVO (Monoart® Euronda, Vicenza, Italy). Evacuators were placed at the level of the tooth around 2 cm from its buccal side.	Yes
107	**Han 2021**	Low speed: 5 min<br>Highspeed: 5s<br>triple syringe: 15s	Air turbine handpiece ((TiMax, NSK, Kanuma, Japan)<br>low-speed air turbine handpiece (W&H implantMED™, Bürmoos, Austria)<br><br>Triple syringe	300,000 rpm<br>1200 rpm	Yes	None	N/A	Yes

36	**Hausler 1966**	not clear	high speed handpiece and air turbine	2.000 rpm	No	No	This is an old study, here is relevance bt the handpieces etc may not be the same	No
108	**Holliday 2021**	10 minutes	Highspeed Hand piece.	**not stated**	Yes	Dental suction at two flow rates: low volume suction, 40 L/min of air; medium volume suction, 159 L/min of air.		No
109	**Ionescu 2020**	4 minutes	* Highspeed: Air turbine handpiece (Bora Led, Bien-Air Dental) equipped with a cylindrical diamond bur (835KR.314.016, Komet Italia Srl).<br><br>**Slow-speed: contra-angle handpiece (CA 1:1, Bien-Air) with a round tungsten carbide bur (H1SM.204.020, Komet)	320,000 rpm.<br>50.000 rpm	Yes	N/A	N/A	Yes

			inside the already prepared cavity.					
91	**Larato 1966**	1.5-5 mins.	air turbine drill	N/A	YES?	Yes	Unit aspirating system	N/A
113	**Llandro 2021**	10 minutes	* speed-increasing dental handpiece driven by a dental air motor at full speed (with no water coolant)		Yes	yes	Large bore dental suction	Yes
50	**Manarte-Monteiro 2013**	1-4 hours	Manual (endodontic) + high-speed handpieces (turbine) (for (endodontic & restorative)	rotary action, with water-cooling;	Yes	Yes	Rubber dam	NA
90	**Miller 1971**	20 seconds	Air turbine Handspeed+ 557 bur (multible procedures). Rubber cup and pumice (periodontics).	N/A	YES?	Yes	High velocity suction	No
115	**Nulty 2020**	1 Minutes	Electric Micromotor: " Micromotor high-speed handpiece with water"Mircomotor<br>Air turbine: HIGH SPEED WITH WATER<br>Low speed with water<br>Three-in-one		Yes	yes	saliva ejector	no
60	**Purohit 2009**	N/A	Prophylaxis was carried out with a Magnetostrictive scaler working at a speed of 30 kHz, with a water	Prophylaxis was carried out with a Magnetostrictive	Yes	No	N/A	Yes

			pressure of 0.3 MPa during each treatment. A high speed air turbine handpiece, working at a speed of 400,000 rpm and with an air drive pressure of 0.25 MPa was used for preparing cavities on carious teeth.	scaler working at a speed of 30 kHz, with a water pressure of 0.3 MPa during each treatment. A high speed air turbine handpiece, working at a speed of 400,000 rpm and with an air drive pressure of 0.25 MPa was used for preparing cavities on carious teeth.				
63	**Rautemaa 2006**	40 minutes	High speed rotating instrument (for the restorative treatment). No equipment details were provided regarding (endodontic/periodontic) treatment	Not stated	N/A	No	N/A	Yes
88	**Samaranayake 1989**	5-15 mins.	Not stated	Not stated	Yes	Not stated	Not stated	No

128	**Shahdad 2021**	20 minutes	air turbine (W&H Synea Turbine TA-98LED, Bürmoos, Austria)	360.000rpm	Yes	Yes	high-volume suction (HVS) and a saliva ejector (SE).	No
94	**Tag El-Din 1997**	5-15 minutes	air-turbine-driven handpiece	Air driven	Yes	Yes	with and without rubber dam	Yes
87	**Yamada 2011**	Not stated	High speed rotating instrument and ultrasonic scaler	Not stated	Yes	Yes	High volume evacuator systems (2 at different locations)	No

Unique Study ID	Study author and reference	If the duration of the procedure was stated, please enter it here	If applicable, what was the main equipment used in performing the procedure? (e.g.rotary handpiece, ultrasonic scaler)	If stated and considered relevant, what was the working mechanism and/or the rotation/vibration speed of the main equipment used	Is the equipment used in performing the dental procedure relevant to today's practice (Please choose from the dropdown list Yes, No, N/A)
99	**Ahmed 2021**	3 minutes	Two-hole AA87handpiece (PANA MAX, NSK, Tochigi, Japan)<br>- four-hole handpiece (KAVO, Biberach, Germany).	**high-speed handpiece**	Yes
100	**Allison 2021(a)**	High Speed Air Turbine = 10mins<br>3-in-1 Spray = 30 seconds (replicate washing acid etchant)	Air turbine - Synea TA-98, W&H (UK) LTD and 3-in-1 Spray = Model not stated	high- speed handpiece. 3-in1 = air and water 140.6mL / min	yes

120	**Allison 2021(b)**	10 minutes	A speed-increasing handpiece (no water) High-speed	**Highspeed:** Approx: 400.000rpm<br><br>Micromotor: 60.000rpm; 120.000rpm; 200.000rpm	yes
127	**Allison 2021(c)**	10 minutes	High-speed air-turbine (Synea TA-98, W&H (UK) Ltd.; St Albans, UK).	**no**	yes
93	**Belting 1964**	1 minute	Air rotor	The dental air rotor (Borden Airotor, Ritter Co., Inc., Rochester, N. Y.) with no. 171 bur attached operating at an air flow pressure of 30 psi using 30 drops of oil and 18ml water per minute	YES?
8	**Bentley 1994**	Experiment 1) 2 minutes (spatter)<br>Experiment 2a) 30 minutes (aerosol with high speed<br>Experiment 2b))30 minutes (aerosol with ultrasonic)	1) high speed handpiece<br>2 high speed handpiece	Not stated	Yes
29	**Grenier 1995**	High-speed drilling= 8 minutes	High speed handpiece	**not stated**	Yes
122	**Grzech-Lesniak 2021**	NA	highspeed handpiece W&H Synea TA-98LC (W&H, Bürmoos, Austria)<br>low-speed handpiece W&H Synea TA-98LC (W&H, Bürmoos, Austria)	**200,000rpm**<br>**15,000rpm**	Yes
107	**Han 2021**	Low speed: 5 min<br>Highspeed: 5s<br>triple syringe: 15s	Air turbine handpiece ((TiMax, NSK, Kanuma, Japan)<br>low-speed air turbine handpiece (W&H implantMED™, Bürmoos, Austria)	**300,000 rpm**<br>1200 rpm	Yes

			Triple syringe		
36	**Hausler 1966**	not clear	high speed handpiece and air turbine	2.000 rpm	No
108	**Holliday 2021**	10 minutes	Highspeed Hand piece.	not stated	Yes
109	**Ionescu 2020**	4 minutes	* Highspeed: Air turbine handpiece (Bora Led, Bien-Air Dental) equipped with a cylindrical diamond bur (835KR.314.016, Komet Italia Srl).<br><br>**Slow-speed: contra-angle handpiece (CA 1:1, Bien-Air) with a round tungsten carbide bur (H1SM.204.020, Komet) inside the already prepared cavity.	**320,000 rpm.**<br>**50.000 rpm**	Yes
91	**Larato 1966**	1.5-5 mins.	air turbine drill	N/A	YES?

113	**Llandro 2021**	10 minutes	* speed-increasing dental handpiece driven by a dental air motor at full speed (with no water coolant)		Yes
50	**Manarte-Monteiro 2013**	1-4 hours	Manual (endodontic) + high-speed handpieces (turbine) (for (endodontic & restorative)	rotary action, with water-cooling;	Yes
90	**Miller 1971**	20 seconds	Air turbine Handspeed+ 557 bur (multible procedures). Rubber cup and pumice (periodontics).	N/A	YES?
115	**Nulty 2020**	1 Minutes	Electric Micromotor: " Micromotor high-speed handpiece with water"Mircomotor<br>Air turbine: HIGH SPEED WITH WATER<br>Low speed with water<br>Three-in-one		Yes
60	**Purohit 2009**	N/A	Prophylaxis was carried out with a Magnetostrictive scaler working at a speed of 30 kHz, with a water pressure of 0.3 MPa during each treatment. A high speed air turbine handpiece, working at a	**Prophylaxis was carried** out with a Magnetostrictive scaler working at a speed of 30 kHz, with a water pressure of 0.3 MPa during each treatment. A high speed air turbine handpiece, working **at a speed of** 400,000 rpm and with an air drive pressure of 0.25 MPa was used for preparing cavities on carious teeth.	Yes

			speed of 400,000 rpm and with an air drive pressure of 0.25 MPa was used for preparing cavities on carious teeth.		
63	**Rautemaa 2006**	40 minutes	High speed rotating instrument (for the restorative treatment). No equipment details were provided regarding (endodontic/periodontic) treatment	Not stated	N/A
88	**Samaranayake 1989**	5-15 mins.	Not stated	Not stated	Yes
128	**Shahdad 2021**	20 minutes	air turbine (W&H Synea Turbine TA-98LED, Bürmoos, Austria)	**360.000rpm**	Yes
94	**Tag El-Din 1997**	5-15 minutes	air-turbine-driven handpiece	**Air driven**	Yes
87	**Yamada 2011**	Not stated	High speed rotating instrument and ultrasonic scaler	Not stated	Yes
